# Composition and Nutritional Quality of the Diet in Spanish Households during the First Wave of the COVID-19 Pandemic

**DOI:** 10.3390/nu13051443

**Published:** 2021-04-24

**Authors:** Susana del Pozo de la Calle, Isabel Alonso Ledesma, Olivier Nuñez, Adela Castelló Pastor, Virginia Lope Carvajal, Nerea Fernández de Larrea Baz, Beatriz Pérez-Gómez, Marina Pollán, Emma Ruiz Moreno

**Affiliations:** 1 Department of Nutrition and Food Science, Complutense University of Madrid, 28040 Madrid, Spain; spozocal@ucm.es; 2 National Centre for Epidemiology, Instituto de Salud Carlos III, 28029 Madrid, Spain; isabel.alonso@isciii.es (I.A.L.); onunez@isciii.es (O.N.); vicarvajal@isciii.es (V.L.C.); nfernandez@isciii.es (N.F.d.L.B.); bperez@isciii.es (B.P.-G.); mpollan@isciii.es (M.P.); 3 Consortium for Biomedical Research in Epidemiology and Public Health, CIBERESP, 28029 Madrid, Spain; 4 School of Medicine, University of Alcalá, 28801 Madrid, Spain; adela.castello@uah.es

**Keywords:** diet, nutritional quality, COVID-19, lockdown

## Abstract

In Spain, the health crisis caused by the COVID-19 pandemic led to the declaration of a state of alarm in the whole country in 2020; in this context, a nationwide lockdown was implemented, potentially altering the dietary habits of the population. The aims of this study were to describe the diet and its nutritional quality in Spanish households during the first COVID-19 epidemic wave and to compare them with the same period in 2019. Data on monthly foods and beverages household purchases in 2019 and 2020 were obtained from the nationwide Food Consumption Surveys. In April, there was an average increase, compared with 2019, of more than 40% for all food groups, with significant peaks in: alcoholic beverages (75%), appetizers (60%), eggs (59%), sugar and sweets (52%), and vegetables (50%). In March, the greatest peak was for pulses, with a 63% increment. The mean energy value of purchased foods in April was 2801 kcal/person/day, corresponding to an increase of 771 kcal/person/day (+38%), compared to the same month of 2019 (March and May: +520 kcal (+26%), June: +343 kcal (+18%)). Regarding nutrient density, there was a reduction in calcium, iodine, zinc, selenium, riboflavin, vitamins B_12_, D, A, especially retinol, and an increase in fibre, sodium, folic acid, carotenes and vitamin E. Alcohol content per 1000 kcal increased by more than 20% from April to July. Food purchase patterns in Spanish households changed during lockdown and after it, with no appreciable improvement in the quality of the diet.

## 1. Introduction

On 11 March 2020, the World Health Organization (WHO) characterized the public health emergency situation caused by COVID-19 disease as a pandemic. This situation began on 31 December 2019, when the Wuhan Municipal Health Commission (Hubei province, China) notified a set of cases of pneumonia of unknown etiology in the city, which were attributed to a new coronavirus, the SARS-CoV-2, shortly afterwards [1].

In Spain, the first case of COVID-19 was diagnosed on the 31 January 2020 [2], and, on March 14, the Spanish Government published Royal Decree 463/2020, declaring a state of alarm for the management of the health crisis, which would be in effect until 21 June 2020 [3].

In the context of the state of alarm, a nationwide lockdown was implemented, including the cessation of all presential education and economic activities, except those considered essentials, such as healthcare, state security, emergencies and food provision. During this period, the population was only allowed to leave home to buy food, hygiene and cleaning products or other basic items, to attend healthcare centres or to provide care for other people [3]. In May, the confinement started to soften, allowing people to go out more frequently and progressively reactivating economic activities.

Dietary habits in Spain have traditionally been characterized by a high consumption of cereals and cereal products, vegetables, fruits, and seafood, a moderate consumption of dairy products and meat, and by the use of olive oil as the main fat source. However, in recent decades, a large part of the Spanish population, mainly the youngest, is moving away from this traditional Mediterranean Diet [4], and a recently published study showed that consumption of cereals and cereal products, milk and dairy products, sugars and sweets, and ready-to-eat meals by children was significantly higher than among adults, while intake of vegetables, fruits, and seafood was higher in adults [5].

In 2020, the new situation in Spain forced people to drastically change their lifestyle, and, as a result, their eating and shopping habits may have been altered. Some studies in Spain found a reduction in the frequency of purchases, but no change in the place where these purchases were made [6], while others detected a higher use of small supermarkets, due to their proximity to homes and the typology of their supplies, with these being the main beneficiaries of the first restriction measures [7]. In other European countries (Germany, Denmark and Slovenia), changes in shopping frequency were also observed; there was a concrete decrease in the shopping frequency for fresh food [8]. Nevertheless, studies published during or shortly after the lockdown, presented conflicting results regarding modifications in dietary habits. Some of them observed an increase in the consumption of healthy foods and in home cooking practices, and a lower consumption of foods of poor nutritional value [7,9], while others found that, during lockdown, diet was characterized by a higher energy intake and lower nutritional quality than in 2019 [10]. Changes in eating habits were also observed in other European countries, such as Italy [11,12,13], Poland [14,15], Denmark [16], Germany, Slovenia [8], Ireland and Great Britain [17]. Although the reasons for these changes are still not fully understood, some authors have suggested that personal characteristics may modulate them, shaping different patterns of change [18,19].

In any case, it seems clear that situations like a severe pandemic can cause changes in eating habits, which could persist over time and have an impact on the health of the population. These changes may vary depending on the degree of restrictiveness and the specific limitations imposed by different confinement strategies. Therefore, it is necessary to assess the magnitude and characteristics of the change in different settings to improve the knowledge of the consequences of lockdown and guide decisions about the most appropriate public health measures. Longitudinal food purchase surveys carried out using a homogeneous methodology over time, and studying representative samples of the population, provide useful information for studying changes over time. Analysis of these data helps overcome some of the main limitations of previous publications, which are mostly based on voluntary cross-sectional web-based surveys, and therefore lack population representativity and are prone to recall biases associated with self-reporting diet changes.

The aims of this study were to describe the diet and its nutritional quality during the first wave of the COVID-19 pandemic in Spain using household food purchase data, and to compare them with the same period of 2019.

## 2. Materials and Methods

### 2.1. Data Source

Data on food and beverages purchases in 2019 and 2020 were obtained from the nationwide representative Food Consumption Survey (FCS) carried out by the Spanish Ministry of Agriculture, Fisheries and Food (MAPA) [20].

The full design and methodology of the survey were described on the MAPA website [21] and elsewhere [22]. Briefly, 12,500 households were selected through a stratified two-stage sampling among all the households in Spain (n = 18,580,014 in 2019 [23]). For this purpose, a household was defined as a person or group of persons who live together in a family home or in part of it and consume food and other assets under a common budget. Households were selected independently of their size or composition. One member of each participating household was responsible for recording, on a daily basis, all food and beverage purchases made for domestic consumption by any member of the household. Products were registered using an optical barcode scanner, except those without a barcode, which were recorded manually using a code book.

### 2.2. Data Processing and Statistical Analysis

Food and beverage data provided by the FCS corresponded to the weight or volume mean of each product purchased by all the participating households (kilos/litters/unit per month per capita). The survey provides aggregated data (mean values) and information on statistical dispersion is not available.

Data corresponding to purchases made from January to August in 2019 and 2020, overall and by region (17 “Autonomous Communities”), were selected. Quantities were converted into grams per person per day. Products were grouped into 15 groups (cereals and cereal products, milk and dairy products, eggs, sugar and sweets, oils and fats, vegetables, pulses, fruits, meat and meat products, fish and seafood, alcoholic beverages, non-alcoholic beverages, sauces and condiments, ready-to-eat-meals, and appetizers/snacks) and 39 subgroups for in depth analyses.

To describe changes in food and beverage purchases between 2019 and 2020, relative percentage changes were calculated as the difference between amounts purchased in a given month in 2020 and 2019, divided by the 2019 amount and multiplied by 100 (% Relative Change = (I_2020_ − I_2019_)/I_2019_* × 100).

To calculate energy value and nutrient content, purchase amounts were codified and transformed using the Food Composition Tables of Moreiras et al., 19th Ed [24], which include more than 800 different foods, and assessed using categorization by groups and subgroups of these tables. For foods whose edible part was not 100%, the inedible portion was discounted [24]. In the case of vegetable oils, a 20% discount was applied to take into account the significant losses they undergo during culinary processes (frying and salad dressing) [25].

Mean energy values were calculated by month for Spain as a whole and for each region. To assess changes in energy values (kcal/person/day) and nutrient content (unit/1000 kcal/person/day) with respect to 2019, the relative percentage of change was calculated, as described above.

Diet quality was evaluated by calculating the following indicators: the percentage of energy obtained from each macronutrient and from alcohol (% energy); the ratios PUFA/SFA, (PUFA + MUFA)/SFA and vitamin E/PUFA; the amounts of cholesterol and fibre; and the nutrient density (nutrient unit/1000 kcal) for 37 nutrients (proteins, carbohydrates, fats, minerals, and vitamins) and alcohol (g/1000 kcal).

Excel 2019 v.2103 software (Microsoft Corporation, California, USA) was used to prepare the database, to code the foods and to estimate energy values and nutrients amounts. Data analyses were performed using Stata MP 15.1 (StataCorp LLC, TX, USA).

## 3. Results

### 3.1. Food and Beverages Groups

Monthly amounts of food and beverages, by food groups, purchased in households during the state of alarm (from March to June 2020) and two months before and after it, are shown in Table 1. As expected, there was an increase in all the food groups, especially during the months of March to June, although a slight increase was observed before the state of alarm (February) and continued, in some food groups, after its end. This increase in the purchase of all food groups can also be observed in the different regions. For example, the purchase of cereals and cereal products in the Balearic Islands (the region with the highest values) in April reached 267 g/person per day, while in January it was 198 g/person per day and in August 162 g/person per day. At the other extreme, cereals and cereal product purchases in La Rioja ranged from 176 g/person per day in April to 137 g/person per day in August.

When comparing these data with the same period in 2019, it should be noted that the highest increases were observed in April, coinciding with the strictest phase of the lockdown. On average, in this month, purchases were a 40% higher than in April 2019, with increases over 50% for eggs, sugar and sweets, vegetables, appetizers, and alcoholic beverages. Across the study period, the highest purchase peak corresponded to alcoholic beverages in April (75% increase), pulses in March (63% increase) and appetizers in April (60%). Purchases in the groups of fish and seafood, milk and dairy products, and ready-to-eat meals showed a slower increase during April and May. Table S1 shows these results by region.

### 3.2. Energy

Table 2 shows the energy content of the purchase in the first eight months of 2020 and the percentage of change from the same months of the previous year, 2019. In accordance with the amount of food purchased, an increase in the energy values was estimated for the months of lockdown. The mean energy value was 2801 kcal/person per day in April 2020, which represents an increase of 771 kcal/person per day (+38%) compared to the same month of 2019, according to household purchases. In March and May, the increase was +528 kcal (+26.2%) and +520 kcal (+26.3%), respectively, and in June, it was +343 kcal (+17.7%).

**Figure 1:**
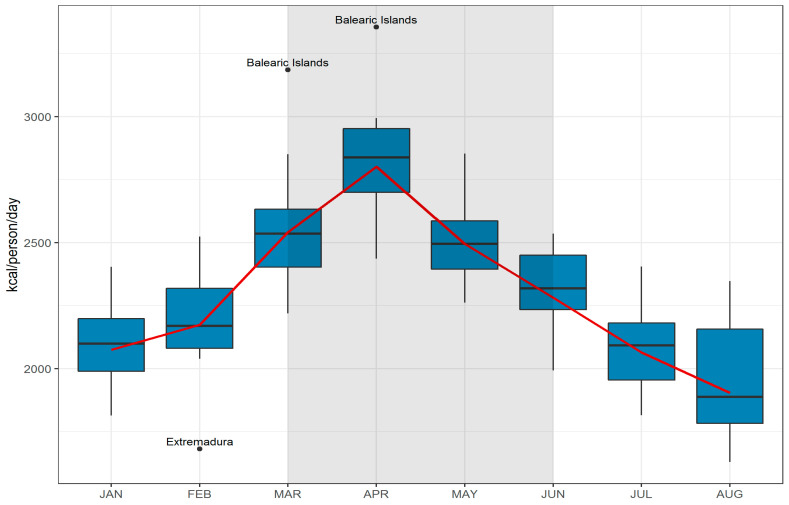
Boxplot of regional variation in mean energy values estimated from food and beverages purchased (kcal/person/day) in households and variation in the national mean (red line), 2020. (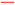) National mean. (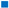) Regions distribution. (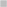) State alarm period (from March 14 to June 21).

The variation in mean energy value of food purchased in Spanish households (kcal/person per day) across regions from January to August 2020 is shown in Figure 1. Consistently with the increase in food purchases observed during lockdown for all the food groups and in all regions, energy value was also increased. The highest average energy value was observed in April, with large differences between regions: the highest value corresponded to the Balearic Islands with 3355 kcal/person per day, and the lowest to La Rioja and Extremadura, with 2436 and 2441 kcal/person per day, respectively. Although April was, for most regions, the month with the largest increase in energy values, there were some regions with a greater increase in March (Balearic Islands and Canary Island (+42%)) or May (Extremadura (+45%)). Table S2 shows the energy estimated from food and beverages purchased in Spanish households, and the relative change with respect to 2019, by region.

In April, cereals and cereal products was the food group that contributed the highest to the energy value (26%), followed by oils and fats, milk and dairy products and meat and meat products (15% each), fruits (7%), and sugar and sweets, vegetables and ready-to-eat-meals (5% each). The remaining groups accounted for less than 3% of the energy value. This pattern was very similar in the other lockdown months, and there were few variations with respect to 2019.

### 3.3. Diet Quality Indexes

Table 3 shows the distribution of macronutrients, sugars, alcohol and fats, expressed as a percentage of the energy value of the purchased foods and beverages in Spanish households in the first eight months of 2020. Overall, no big differences were observed in the studied period in the distribution of energy sources. Alcohol and saturated fatty acids rose slightly in April and remained increased until August. Over the study period, carbohydrates were at the lower limit of the EFSA recommendations for 2019, while fats were well above these recommendations [26].

Regarding compliance with the nutritional goal for fibre in the Spanish population, the increase estimated during lockdown (March 22.4 g, April 24.7 g, May 21.3 g and June 18.8 g), did not reach the recommendation for the adult population (>25 g/day) [26], although it improved in comparison to the average value of the year 2019 (16.6 g/day).

In relation to other fat quality indexes, the estimated amount of daily cholesterol in the diet of these months (March 404 mg, April 468 mg, May 411 mg and June 375 mg) was over the recommendations (<300 mg/day) [27]. The ratios PUFA/SFA and (PUFA + MUFA)/SFA showed values above 0.5 and 2.0, respectively, in all the months studied, except July ((PUFA + MUFA)/SFA: 1.99). The highest values were observed in March, reaching 0.67 and 2.11, respectively. In the same way, the vitamin E/PUFA ratio was within the recommendations during all the studied period, with an average value of 0.59 [24,27].

Along with an increase in the purchases of all the food groups, there was an increase in the absolute energy and nutrient values. With respect to nutrient density (amount of nutrient per 1000 kcal), the results were diverse. Compared to 2019, during the first eight months of 2020 (Table 4), a reduction was seen for calcium, iodine, zinc, selenium, riboflavin, vitamin B_12_, vitamin D and vitamin A (especially retinol). Conversely, there was an increase in fibre, sodium, folic acid, carotenes and vitamin E density. Alcohol content per 1000 kcal increased by more than 20% with respect to 2019 from April to July.

## 4. Discussion

In our study, we observed a change in the food purchase pattern in Spanish households during the state of alarm period (14 March to 21 June 2020) compared to the same months of 2019. These changes, characterized by an increase in purchases of all the food groups, took place mainly in April and May, corresponding to the strictest lockdown in Spain, although pulses and oils purchases rose in February, and some changes were still observed after June. However, overall purchases did not return to the values of 2019 in any of the months studied. In terms of quality of the diet, there were no differences in macronutrient distribution, and nutrient density increased for some nutrients while it decreased for others.

As expected, from March to June 2020, with respect to 2019, there was an increase in the purchase of all food groups, overall and by region. In other European countries, such as Denmark, Slovenia or Germany, this increase was not observed in all food groups [8].

Between February and August 2020, a decrease in the purchase of non-perishable products is observed, but this decrease was not linear, and instead, there was an increase during the months of lockdown. On the other hand, in the perishable food groups such as vegetables, fish or eggs, there was also an increase during the lockdown, but the decrease between February and August was not so marked. This difference may be due to several factors, including the higher frequency of meals made at home due to restaurants’ closure during the lockdown, and the compulsive purchase and stockpiling of non-perishable foods such as pulses, canned food, bottled water or beer due to the fear of food shortages at the beginning of the pandemic. The different behaviours in the purchase of perishable and non-perishable products could represent a consequence of the excess of non-perishable products purchased during the lockdown and the confinement softening phase, which had still not been consumed at the end of the study period, rendering their purchase less necessary than that of perishable foods. A different pattern of fresh and non-fresh food shopping was observed in other European countries, such as Germany, Denmark, Slovenia and Italy, where a greater decrease was observed in the purchase of fresh products compared to non-fresh products [8,28]. Nevertheless, seasonal trends in dietary patterns may also have contributed to this finding.

As the lockdown period advanced, sales of products related to home baking, such as flour or yeast, significantly increased in supermarkets (+147%), as highlighted in the ”Balance of Distribution and Mass Consumption in Spain” study carried out by Kantar, a data and consulting company [29]. In line with these observations, during the lockdown, the consumption of desserts and homemade cakes increased [30]. Concretely, in the week when the state of alarm was declared in Spain, March 9 to 15, purchases increased by 21% [7].

In the case of appetizers and alcoholic and non-alcoholic beverages, their purchases increased during lockdown, with respect to the same months of 2019, and remained high until August. Appetizer purchases nearly doubled between January (12.8 g/person/day) and April 2020 (22.1 g/person/day), in contrast to the same period of 2019, when they remained constant (January 12.2 g and April 13.8 g/person/day). An increase in the consumption of appetizers was also observed in other Spanish studies carried out during the lockdown [19], as well as in other countries such as Poland [31], Italy [12] or France [32], although other studies found no change or even decreases [33]. This increment in the purchase of appetizers is probably due to the desire to continue with some socialization habits that people did outdoors before the pandemic [28]. These changes were consistently observed in different regions, with small differences among them.

There are big differences in food consumption patterns between 2019 and 2020, with a high reduction in the consumption of food and beverages in bars and restaurants as a consequence of movement and economic activity restrictions and fear of being infected. Therefore, to evaluate changes in population habits between these years, the contribution of food consumption outside homes to the global diet should be considered. According to the limited data published by the FCS on extra-domestic consumption, in 2019, the sum of household plus extra-domestic purchases was still lower than household purchases during lockdown in 2020. It can be highlighted, for example, that household food purchases in April 2020 were 30–40% higher than the sum of household and extra-domestic purchases in April 2019 for food groups such as sugars and sweets, oils and fats, fruits, vegetables and appetizers, and more than 65% higher for eggs, and sauces and condiments [34].

With respect to alcoholic beverages, household purchases during the first state of alarm in 2020, were 30% lower than the sum of household and extra-domestic purchases during the same period in 2019. Reductions in alcohol consumption, were also found in other studies [18,33]. In the context of the recently updated definition of the limits for low-risk alcohol consumption (20 g/day for men and 10 g/day for women) in Spain [35], the average amounts purchased during the lockdown were under these limits (March 4.94 g, April 7.58 g and May 7.04 g and June 6.19 g), although they were much higher than the 2019 average household purchases (4.37 g/p/d). However, even if the total alcohol purchases diminished, monitoring future trends in household purchases of alcoholic beverages would be important, in order to avoid the purchase of alcoholic beverages for consumption at home becoming a new habit, which could lead to increases in alcohol consumption over time.

Changes in food purchase and consumption habits during the COVID-19 pandemic have also been observed in other European countries, although the results are heterogeneous. Some of the studies report improvements and others report worsening diets. In Italy, the lockdown highly affected food choice behaviours, leading to positive and sustainable purchase and consumption habits [13,36]. In the PLACE-19 study, in Polish adolescents, the authors concluded that the COVID-19 pandemic may have changed the food choice determinants, increasing the importance of health and weight control while reducing the role of mood and sensory appeal [15]. These may be interpreted as positive changes promoting a healthy diet. On the other hand, other studies found more ambiguous results. Increased intake of fruits, pulses and fish, and diminished consumption of sweets, grains, nuts and dairy products, but no changes in overall adherence to the MD, were observed among students in Croatia [37]. In another survey, carried out in several English-speaking countries (Island of Ireland, Great Britain, United States, and New Zealand), positive cooking-related practices and increases in fruit and vegetable intake were found, while an increase in saturated fat intake was also observed [17]. A limitation of self-reported data about perceived changes in the quality of the diet is that they depend on participants’ nutritional knowledge, and the perception of a better diet does not necessarily imply an improvement in nutritional quality.

The assessment of the energy and nutrient content of the food and beverage purchases made by the Spanish population, overall and by region, showed that there was a significant increase in the energy content during lockdown, reaching a general increment of 38% in April 2020 compared to 2019. An average energy value of 2801 kcal/person per day was estimated during the month of April 2020, which represents an increase of 771 kcal/person per day. Likewise, although to a lesser extent, in the other months of lockdown, there was also an increase in energy values. Even though food purchases do not equal food intake, mainly due to food waste, it has been reported that household food purchases, when collected exhaustively, are a reasonably accurate estimate of diet quality [38]. On the other hand, it is also important to highlight that the energy needs of the population during lockdown were, on average, lower than in 2019, due to people having lower mobility and a reduction in physical activity [30,39]. If we compare April 2020 with the average energy values of previous years in Spain, we can see that this figure is close to those of the Spanish population from 1981 to 2006 (1981: 2914 kcal/person/day; 2000: 2730 kcal/person/day; 2006: 2761 kcal/person/day), but it is much higher than in more recent years (2009: 2217 kcal/person/day; 2011: 2075 kcal/person/day) [4,40]. Other studies have estimated that the average energy intake in Spain in 2013 was 1810 kcal/person per day, taking into account extra-domestic consumption and food waste [41]. To our knowledge, few studies have assessed changes in energy values during the pandemic. In the NutriNet-Santé cohort, a reduction in energy values was found with respect to estimates from 2017 to 2019 [32]. More studies would be needed to assess whether there are some common patterns associated with lockdowns, or, on the contrary, whether changes are country- or even region-specific.

In relation to the quality of the diet, macronutrients’ distribution, expressed as percentage of the energy, showed, in all the months studied, a high contribution of fats, at the expense of carbohydrates. No remarkable change was observed associated with the lockdown. In Spain, the percentage contribution of carbohydrates has been decreasing steadily since 1964, when it was in line with the recommendations [4].

With respect to dietary fat quality indicators such as PUFA/SFA and (PUFA + MUFA)/SFA ratios, a positive aspect that should be maintained was the high proportion of MUFA, due to the common consumption of olive oil in Spain [42]. On the other hand, SFA seemed to rise slightly during the studied period. The PUFA/SFA and (PUFA + MUFA)/SFA relationships show an improvement during the month of March, 0.67 and 2.11 respectively, compared to March 2019, when they were 0.65 and 2.04, respectively.

As regards as nutrient density (nutrient amount/1000 kcal), it decreased for some nutrients while it improved for others. If we compare the data obtained in 2020 with the year 2019, nutrient density was reduced for calcium, iodine, zinc, selenium, riboflavin, vitamin B_12_, vitamin D and vitamin A, especially retinol. On the contrary, the density of fibre, sodium, folic acid, carotenes and vitamin E increased. In the case of alcohol content per 1000 kcal, a remarkable 20% increase started in April and remained until July. Among the nutrients whose density decreased, vitamin D should be highlighted, as due to the reduced sun exposure the population faces during lockdown, the risk of vitamin D deficiency could be increased, and low levels of this vitamin have been related to several adverse health consequences, including more severe COVID-19 [43,44].

The limitations of this research include the use of a secondary data source, with only aggregated data (mean values) freely available, lacking information on statistical dispersion and uncertainty. The lack of information at the individual level prevents the realization of analyses that account for other variables that could have an influence on dietary changes. It is also important to highlight that the analysed data correspond exclusively to household purchases, and, therefore, extra-domestic consumption, e.g., meals in restaurants, was not considered. Unfortunately, the data available about extra-domestic consumption in Spain are gathered using a different methodology and cannot be directly accumulated to household purchase data. Lastly, our estimates of energy values and nutrient density have not taken food waste into account, and could, therefore, overestimate real values.

Our study has also some strengths, notably that it is based on data collected from a nationwide representative sample of the Spanish population, collected following a methodology that has been established for decades. Additionally, the prospective nature of the FCS, gathering purchase data provided by a rather constant group of households across the years, allowed us to analyse data that were collected before the pandemic, overcoming biases associated with the subjective perception of changes in the diet. Likewise, we were able to make month-by-month comparisons between purchases before and during the pandemic, reducing the impact that seasonal variability in dietary habits may have when comparing different months. Lastly, we analysed the change in the nutritional quality of the diet, providing relevant information that is still not widely available in relation to the COVID-19 pandemic.

## 5. Conclusions

Food purchase patterns in Spanish households changed greatly during the months of lockdown and, to a lesser extent, afterwards, with an increase in the purchase of all food groups, especially alcoholic beverages, appetizers, eggs, sugar and sweets, vegetables and pulses. In relation to macronutrient distribution, sugars, alcohol, and fats, expressed as percentage of the energy, did not vary with respect to 2019. Nutrient density showed both favourable and unfavourable changes, depending on the specific nutrient.

According to our results and those published by other authors, the COVID-19 pandemic and the restrictions implemented by most countries to control it have been associated with dietary changes in the population. Although heterogeneous results have been reported from different countries when analyzing specific food groups, most of them found that a significant proportion of the population increased food purchases or overall intake. Our results, based on exhaustive, prospectively collected purchase data, do not support the hypothesis of an improvement in the quality of diet during lockdown compared to the same period of 2019.

Therefore, it is necessary to continue with the surveillance of the quality of diet during the pandemic, especially through the different epidemic waves and lockdown scenarios, in order to quickly detect possible deleterious dietary changes and establish preventive measures accordingly. In this sense, it would be very important to deepen into our understanding of the factors associated with negative and positive changes in diet, to identify groups more prone to acquiring unhealthy habits, and to promote factors associated with positive dietary changes.

## Figures and Tables

**Table 1:** Food and beverage purchases in Spanish households in 2020 (grams/person/day), and relative change with respect to 2019 (%).

	JAN		FEB		MAR		APR		MAY		JUN		JUL		AUG	
PRODUCTS (g/p/Day)	2020	Change (%)	2020	Change (%)	2020	Change (%)	2020	Change (%)	2020	Change (%)	2020	Change (%)	2020	Change (%)	2020	Change (%)
Cereals and cereal products	163.3	2.9	172.4	5.4	199.9	24.4	214.8	34.3	185.9	20.8	166.1	11.8	148.8	5.6	139.3	3.0
Milk and dairy products	297.1	1.0	306.9	2.9	350.9	20.1	373.1	26.3	341.2	16.3	318.5	12.6	299.2	9.7	270.8	2.0
Eggs	24.5	8.9	25.4	9.6	29.8	26.5	37.8	59.3	31.5	38.9	27.5	22.3	25.7	16.1	23.8	10.9
Sugar and sweets	25.2	1.5	23.3	2.1	30.1	30.6	36.1	52.4	27.8	34.9	23.5	24.9	18.8	13.6	17.5	1.4
Oils and fats	29.3	6.0	31.6	14.1	38.0	36.5	39.4	33.0	36.8	29.8	34.2	24.4	29.1	11.2	28.7	11.8
Vegetables	247.4	2.9	275.5	9.3	313.0	25.7	364.9	50.4	329.9	32.0	292.6	18.9	280.3	9.2	247.4	2.2
Pulses	14.2	5.3	14.0	12.0	20.0	62.7	16.9	48.8	13.6	32.1	12.0	20.9	10.0	12.8	9.8	16.0
Fruits	241.9	1.6	267.9	6.3	293.9	14.0	354.7	40.3	337.0	22.3	334.5	12.2	338.4	8.3	299.8	7.6
Meat and meat products	132.5	1.5	136.3	4.4	155.7	21.2	169.6	36.1	151.3	24.3	139.7	19.7	123.1	13.4	112.2	5.5
Fish and seafood	58.3	−1.5	62.7	2.9	67.8	9.1	80.1	28.0	76.9	29.8	70.3	16.6	66.1	12.3	59.3	8.2
Alcoholic Beverages	64.4	4.5	72.4	7.9	86.6	20.5	127.7	75.2	123.4	64.8	110.8	36.4	110.0	35.6	95.5	18.9
Non-alcoholic Beverages	295.6	0.7	321.3	5.1	354.1	12.9	396.7	28.9	402.4	23.3	399.0	14.0	410.6	8.5	393.7	9.7
Sauces and condiments	29.4	8.5	29.3	6.8	36.5	30.3	40.3	46.1	36.8	31.6	33.1	22.2	30.1	11.1	28.2	8.0
Ready-to-eat-meals	49.6	9.1	48.2	14.6	53.2	31.8	52.3	28.9	47.4	16.3	48.4	15.9	45.9	10.0	40.5	2.2
Appetizers	12.8	4.4	14.1	10.8	16.4	26.5	22.1	60.3	20.0	47.5	18.1	28.8	16.8	17.2	14.9	10.7

(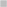) State of alarm period (from March 14 to June 21).

**Table 2:** Energy values estimated from food and beverages purchased in Spanish households in 2020 (kcal/person/day), and relative change with respect to 2019 (%).

	JAN	FEB	MAR	APR	MAY	JUN	JUL	AUG
Total 2020	2074	2174	2542	2801	2495	2281	2065	1904
Vs. 2019 (%)	+3.9	+7.2	+26.2	+38.0	+26.3	+17.7	+10.4	+5.9

(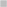) State of alarm period (from March 14 to June 21).

**Table 3:** Macronutrients and alcohol distribution estimated from food and beverages purchased in Spanish households in 2020 (% of the energy).

	JAN	FEB	MAR	APR	MAY	JUN	JUL	AUG
**Proteins (%)**	15.1	15.0	14.7	14.6	14.7	14.7	14.8	14.6
**Fats (%)**	39.7	39.8	39.6	39.8	40.4	40.7	40.1	40.6
SFA (%)	11.5	11.6	11.4	11.8	11.9	11.9	11.9	12.0
MUFA (%)	16.6	16.6	16.4	16.2	16.8	17.2	16.6	16.9
PUFA(%)	7.4	7.4	7.6	7.7	7.5	7.3	7.1	7.4
**Carbohydrates (%)**	42.2	42.1	42.6	42.0	41.2	41.0	41.6	41.4
Sugars (%)	16.6	16.6	16.4	17.0	17.2	17.4	17.9	17.7
**Alcohol (%)**	1.3	1.4	1.4	1.9	2.0	1.9	2.0	1.8

SFA: Saturated fatty acids, MUFA: Monounsaturated fatty acids, PUFA: Polyunsaturated fatty acids. (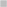) State of alarm period.

**Table 4:** Relative change in nutrient density (unit/1000 kcal) and alcohol (g/1000 kcal) estimated from food and beverages purchased in Spanish households in 2020 with respect to 2019 (%).

	JAN	FEB	MAR	APR	MAY	JUN	JUL	AUG
Proteins	−0.61	−1.17	−2.49	−1.74	−1.28	−0.49	0.34	−0.65
Carbohydrates	−0.79	−1.30	0.58	−0.06	−2.19	−2.91	−2.34	−1.98
Starch	−0.23	−0.46	4.38	0.44	−1.91	−2.58	−2.65	−1.76
Sugars	−1.66	−2.55	−4.95	−0.78	−2.58	−3.37	−1.91	−2.28
Fibre	0.28	0.84	0.99	3.03	0.61	−1.49	−1.80	−1.28
Fats	1.20	1.84	0.63	−0.51	1.60	2.56	1.53	1.84
SFA	0.84	0.57	−1.68	0.39	1.55	1.53	2.07	1.13
MUFA	2.28	3.99	1.66	−1.92	2.16	4.50	2.20	2.80
PUFA	−1.00	−1.01	1.71	0.69	0.57	0.23	−1.06	2.07
Cholesterol	0.31	−1.10	−5.34	0.18	0.05	0.36	1.44	−0.52
Oleic Acid	−0.97	0.99	2.54	0.47	−0.14	2.80	1.57	5.26
w-3	−2.85	−2.85	2.92	2.38	−0.76	−0.67	−2.36	2.76
w-6	−4.47	−2.15	−3.50	−2.51	1.17	−0.16	2.34	1.30
C20:5 (EPA)	−4.89	−4.08	−5.33	−8.34	5.33	−1.14	3.04	1.96
C22:6 (DHA)	−7.27	−5.23	−5.98	−9.44	4.38	−2.68	1.97	0.61
	**Alcohol**							
	−3.35	−0.10	−7.80	29.63	31.13	19.84	23.29	13.61
	**Minerals**							
Calcium	0.04	−1.60	−3.64	−2.51	−1.97	−1.38	0.05	−2.00
Iron	−0.30	−0.45	−2.19	−0.12	0.37	0.00	−0.04	−0.45
Iodine	−3.50	−3.68	−1.60	−6.55	−6.33	−1.95	0.41	−3.68
Magnesium	−0.15	0.13	−0.73	0.68	−0.08	0.09	0.25	0.00
Zinc	−0.05	−0.89	−3.65	−2.08	−1.92	−1.32	−0.56	−1.25
Sodium	−1.36	0.11	1.96	3.06	−0.81	0.69	−3.11	2.00
Potassium	−1.24	−0.22	−1.66	1.09	0.06	−0.14	0.19	−1.30
Phosphorus	2.18	−0.08	−1.95	0.83	0.75	0.12	0.83	0.05
Selenium	−1.34	−1.53	−3.51	−3.96	−2.11	−3.05	−1.31	−0.70
	**Vitamins**							
Thiamine	−1.21	−0.77	−1.70	0.53	−1.38	−0.76	−0.27	−0.48
Riboflavin	−1.11	−2.02	−4.63	−2.60	−2.50	−1.03	0.79	−1.70
Niacin	−0.48	−0.77	−2.01	−1.87	−0.82	0.26	1.24	−0.37
Vitamin B_6_	−1.32	−0.45	−1.45	0.68	−0.22	−0.12	0.89	0.03
Folates	0.01	−0.07	−0.95	3.97	1.66	−0.96	−0.66	−0.79
Vitamin B_12_	0.17	−2.89	−5.74	−6.27	−2.45	−1.66	2.59	−0.69
Vitamin C	−1.37	−0.65	−6.94	3.40	0.60	−2.20	−1.10	−1.64
Vitamin A	−2.40	−9.73	−7.24	−4.02	1.40	−0.63	3.84	−2.28
Retinol	−4.45	−15.53	−10.73	−12.50	−3.28	−2.54	7.36	−4.49
Carotenes	0.32	−0.48	−2.54	8.08	7.26	1.13	0.00	−0.03
Vitamin D	−2.45	−1.28	−3.66	−8.78	2.57	−3.02	2.14	−0.02
Vitamin E	−0.34	−0.10	1.67	2.10	2.41	0.63	−2.46	1.64

Colour gradient, from (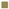) highest to (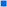) lowest density. (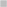) State of alarm period. SFA: Saturated fatty acids, MUFA: Monounsaturated fatty acids, PUFA: Polyunsaturated fatty acids. EPA: Eicosapentaenoic acid, DHA: Docosahexaenoic acid.

## Data Availability

Data are available in: Ministerio de Agricultura, Pesca y Alimentación. Panel de Consumo Alimentario. Available online: https://www.mapa.gob.es/es/alimentacion/temas/consumo-tendencias/panel-de-consumo-alimentario/ (accessed on 10 December 2020).

## Supplementary Materials

The following are available online at https://www.mdpi.com/article/10.3390/nu13051443/s1, Table S1: Food and beverages purchase in Spanish households by regions in 2020 (grams/person/day), and relative change with respect to 2019 (%). Table S2: Energy values estimated from food and beverages purchased in Spanish households by region in 2020 (kcal/person/day), and relative change with respect to 2019 (%).

## References

[1] World Health Organization (WHO) Listings of WHO’s Response to COVID-19. https://www.who.int/news/item/29-06-2020-covidtimeline.

[2] Centro Nacional de Epidemiología Instituto de Salud Carlos III Informe COVID-2019 No 1_11 de Febrero de 2020. Primeros Casos Investigados en España por COVID-2019. https://www.isciii.es/QueHacemos/Servicios/VigilanciaSaludPublicaRENAVE/EnfermedadesTransmisibles/Documents/INFORMES/InformesCOVID-19/InformeCOVID-19.No1_11febrero2020_ISCIII.pdf.

[3] Agencia Estatal Boletín Oficial del Estado Real Decreto 463/2020, de 14 de Marzo, por el Que Se Declara el Estado de Alarma Para la Gestión de la Situación de Crisis Sanitaria Ocasionada por el COVID-19. https://www.boe.es/buscar/act.php?id=BOE-A-2020-3692.

[4] Varela-Moreiras G., Ruiz E., Valero T., Ávila J.M., Del Pozo S. (2013). The Spanish diet: An update. Nutr. Hosp..

[5] Partearroyo T., Samaniego-Vaesken M.D.L., Ruiz E., Aranceta-Bartrina J., Gil Á., González-Gross M., Ortega R.M., Serra-Majem L., Varela-Moreiras G. (2019). Current food consumption amongst the spanish anibes study population. Nutrients.

[6] Laguna L., Fiszman S., Puerta P., Chaya C., Tárrega A. (2020). The impact of COVID-19 lockdown on food priorities. Results from a preliminary study using social media and an online survey with Spanish consumers. Food Qual. Prefer..

[7] Valencoso C. La Declaración del Estado de Alarma Dispara la Compra. https://www.kantar.com/es/inspiracion/coronavirus/la-declaracion-del-estado-de-alarma-dispara-la-compra.

[8] Janssen M., Chang B.P.I., Hristov H., Pravst I., Profeta A., Millard J. (2021). Changes in Food Consumption During the COVID-19 Pandemic: Analysis of Consumer Survey Data From the First Lockdown Period in Denmark, Germany, and Slovenia. Front. Nutr..

[9] Pérez-Rodrigo C., Citores M.G., Hervás Bárbara G., Litago F.R., Casis Sáenz L., Aranceta-Bartrina J., Val V.A., López-Sobaler A.M., Martínez De Victoria E., Ortega R.M. (2020). Cambios en los hábitos alimentarios durante el periodo de confinamiento por la pandemia COVID-19 en España. Rev. Esp. Nutr. Comunitar..

[10] Batlle-Bayer L., Aldaco R., Bala A., Puig R., Laso J., Margallo M., Vázquez-Rowe I., Antó J.M., Fullana-i-Palmer P. (2020). Environmental and nutritional impacts of dietary changes in Spain during the COVID-19 lockdown. Sci. Total Environ..

[11] Brancaccio M., Mennitti C., Gentile A., Correale L., Buzzachera C.F., Ferraris C., Montomoli C., Frisso G., Borrelli P., Scudiero O. (2021). Effects of the covid-19 pandemic on job activity, dietary behaviours and physical activity habits of university population of Naples, federico ii-Italy. Int. J. Environ. Res. Public Health.

[12] Fanelli R.M. (2021). Changes in the Food-Related Behaviour of Italian Consumers during the COVID-19 Pandemic. Foods.

[13] Scacchi A., Catozzi D., Boietti E., Bert F., Siliquini R. (2021). COVID-19 Lockdown and Self-Perceived Changes of Food Choice, Waste, Impulse Buying and Their Determinants in Italy: QuarantEat, a Cross-Sectional Study. Foods.

[14] Kowalczuk I., Gębski J. (2021). Impact of Fear of Contracting COVID-19 and Complying with the Rules of Isolation on Nutritional Behaviors of Polish Adults. Int. J. Environ. Res. Public Health.

[15] Głąbska D., Skolmowska D., Guzek D. (2020). Population-based study of the changes in the food choice determinants of secondary school students: Polish adolescents’ COVID-19 experience (place-19) study. Nutrients.

[16] Giacalone D., Frøst M.B., Rodríguez-Pérez C. (2020). Reported Changes in Dietary Habits During the COVID-19 Lockdown in the Danish Population: The Danish COVIDiet Study. Front. Nutr..

[17] Murphy B., Benson T., McCloat A., Mooney E., Elliott C., Dean M., Lavelle F. (2021). Changes in consumers’ food practices during the covid-19 lockdown, implications for diet quality and the food system: A cross-continental comparison. Nutrients.

[18] Ammar A., Brach M., Trabelsi K., Chtourou H., Boukhris O., Masmoudi L., Bouaziz B., Bentlage E., How D., Ahmed M. (2020). Effects of COVID-19 home confinement on eating behaviour and physical activity: Results of the ECLB-COVID19 international online survey. Nutrients.

[19] Pérez-Rodrigo C., Gianzo Citores M., Hervás Bárbara G., Ruiz-Litago F., Casis Sáenz L., Arija V., López-Sobaler A.M., Martínez de Victoria E., Ortega R.M., Partearroyo T. (2021). Patterns of Change in Dietary Habits and Physical Activity during Lockdown in Spain Due to the COVID-19 Pandemic. Nutrients.

[20] Ministerio de Agricultura, Pesca y Alimentación Panel de Consumo Alimentario. https://www.mapa.gob.es/es/alimentacion/temas/consumo-tendencias/panel-de-consumo-alimentario/.

[21] Ministerio de Agricultura, Pesca y Alimentación Panel de Consumo Alimentario. Metodología. https://www.mapa.gob.es/es/alimentacion/temas/consumo-tendencias/panel-de-consumo-alimentario/metodologia/default.aspx.

[22] del Pozo de la Calle S., Ruiz Moreno E., Valreo Gaspar T., Rodríguez Alonso P., Ávila Torres J.M. (2015). Sources of information on food consumption in Spain and Europe. Nutr. Hosp..

[23] Instituto Nacional de Estadística Proyeccion de Hogares. https://www.ine.es/dyngs/INEbase/es/operacion.htm?c=Estadistica_C&cid=1254736176954&menu=ultiDatos&idp=1254735572981.

[24] Moreiras O., Carbajal Á., Cabrera L., Cuadrado C. (2018). Tablas de Composición de Alimentos.

[25] Varela Mosquera G. (1993). Posibilidades de la fritura de los alimentos en la relación dieta/enfermedades degenerativas. Rev. Sanid. Hig. Publica.

[26] (2017). Dietary Reference Values for nutrients Summary report. EFSA Support. Publ..

[27] Serra Majem L. (2011). Objetivos nutricionales para la población española: Consenso de la Sociedad Española de Nutrición Comunitaria 2011. Rev. Española Nutr. Comunitaria Span. J. Community Nutr..

[28] Bracale R., Vaccaro C.M. (2020). Changes in food choice following restrictive measures due to Covid-19. Nutr. Metab. Cardiovasc. Dis..

[29] García F. El Desconfinamiento Revoluciona la Distribución. https://www.kantarworldpanel.com/es/Noticias/El-desconfinamiento-revoluciona-la-distribucin.

[30] Sánchez-Sánchez E., Ramírez-Vargas G., Avellaneda-López Y., Orellana-Pecino J.I., García-Marín E., Díaz-Jimenez J. (2020). Eating habits and physical activity of the spanish population during the covid-19 pandemic period. Nutrients.

[31] Sidor A., Rzymski P. (2020). Dietary Choices and Habits during COVID-19 Lockdown: Experience from Poland. Nutrients.

[32] Deschasaux-Tanguy M., Druesne-Pecollo N., Esseddik Y., de Edelenyi F.S., Allès B., Andreeva V.A., Baudry J., Charreire H., Deschamps V., Egnell M. (2021). Diet and physical activity during the coronavirus disease 2019 (COVID-19) lockdown (March–May 2020): Results from the French NutriNet-Santé cohort study. Am. J. Clin. Nutr..

[33] Poelman M.P., Gillebaart M., Schlinkert C., Dijkstra S.C., Derksen E., Mensink F., Hermans R.C.J., Aardening P., de Ridder D., de Vet E. (2021). Eating behavior and food purchases during the COVID-19 lockdown: A cross-sectional study among adults in the Netherlands. Appetite.

[34] Ministerio de Agricultura Pesca y Alimentación Consumo Extradoméstico por Tipo de Alimento. https://www.mapa.gob.es/es/alimentacion/temas/consumo-tendencias/panel-de-consumo-alimentario/hosteleria-y-restauracion/.

[35] Sordo L., Córdoba R., Gual A., Sureda X. (2020). Límites para el consumo de bajo riesgo de alcohol en función de la mortalidad asociada. Rev. Esp. Salud Publica.

[36] Di Renzo L., Gualtieri P., Pivari F., Soldati L., Attinà A., Cinelli G., Cinelli G., Leggeri C., Caparello G., Barrea L. (2020). Eating habits and lifestyle changes during COVID-19 lockdown: An Italian survey. J. Transl. Med..

[37] Dragun R., Veček N.N., Marendić M., Pribisalić A., Ðivić G., Cena H., Polašek O., Kolčić I. (2021). Have lifestyle habits and psychological well-being changed among adolescents and medical students due to COVID-19 lockdown in Croatia?. Nutrients.

[38] Appelhans B.M., French S.A., Tangney C.C., Powell L.M., Wang Y. (2017). To what extent do food purchases reflect shoppers’ diet quality and nutrient intake?. Int. J. Behav. Nutr. Phys. Act..

[39] Martinez-Ferran M., de la Guía-Galipienso F., Sanchis-Gomar F., Pareja-Galeano H. (2020). Metabolic impacts of confinement during the COVID-19 pandemic due to modified diet and physical activity habits. Nutrients.

[40] Varela Moreiras G., Del Pozo S., Ávila Torres J.M., Cuadrado Vives C., Ruiz Moreno E., Moreiras Tuny O. (2009). Valoración de la dieta española de acuerdo al Panel de Consumo Alimentario. Distrib. Consumo.

[41] Ruiz E., Ávila J.M., Valero T., del Pozo S., Rodriguez P., Aranceta-Bartrina J., Gil Á., González-Gross M., Ortega R.M., Serra-Majem L. (2015). Energy Intake, Profile, and Dietary Sources in the Spanish Population: Findings of the ANIBES Study. Nutrients.

[42] Aranceta J. (2001). Spanish food patterns. Public Health Nutr..

[43] Liu N., Sun J., Wang X., Zhang T., Zhao M., Li H. (2021). Low vitamin D status is associated with coronavirus disease 2019 outcomes: A systematic review and meta-analysis. Int. J. Infect. Dis..

[44] Ilie P.C., Stefanescu S., Smith L. (2020). The role of vitamin D in the prevention of coronavirus disease 2019 infection and mortality. Aging Clin. Exp. Res..

