# Supplementary material for: Composition and Nutritional Quality of the Diet in Spanish Households during the First Wave of the COVID-19 Pandemic
Source: Nutrients. 2021 Apr 24;13(5):1443. doi: 10.3390/nu13051443 (PMC8146770; doi:10.3390/nu13051443)
Supplement: Supplementary file 1 [file nutrients-13-01443-s001.zip › nutrients-1142819-SI.pdf]

Table S1. Food groups consumption by regions. Year 2020 and change versus 2019.

JANUARY	CATALONIA		ARAGON		BALEARIC ISLANDS		VALENCIAN COMMUNITY		MURCIA		ANDALUSIA		COMMUNITY OF MADRID		CASTILE LA MANCHA	
(g/p/day)	2020	*Change (%)*	2020	*Change (%)*	2020	*Change (%)*	2020	*Change (%)*	2020	*Change (%)*	2020	*Change (%)*	2020	*Change (%)*	2020	*Change (%)*
**Cereals and derivatives**	161,8	*1,7*	141,9	*4,1*	189,0	*9,1*	172,0	*4,5*	155,4	*6,1*	154,3	*6,9*	147,3	*4,4*	159,1	*-8,4*
**Milk and dairy products**	281,6	*1,7*	275,6	*1,8*	298,9	*13,3*	271,4	*4,6*	272,0	*-2,3*	262,0	*4,1*	304,0	*2,5*	311,8	*-1,1*
**Eggs**	24,9	*10,0*	30,6	*32,4*	23,8	*-3,9*	24,7	*11,6*	19,9	*-13,8*	22,5	*11,0*	24,8	*8,8*	23,8	*10,8*
**Sugar and sweets**	22,9	*4,6*	22,7	*5,8*	28,9	*20,1*	26,4	*8,4*	23,0	*-7,0*	22,0	*3,4*	23,4	*-2,4*	25,0	*-0,5*
**Oils and fats**	29,5	*0,5*	34,9	*41,2*	37,3	*12,8*	24,4	*16,3*	16,1	*-21,6*	27,4	*8,2*	25,6	*7,5*	20,9	*-30,5*
**Vegetables**	304,2	*7,9*	273,2	*3,9*	271,1	*5,3*	266,8	*2,6*	237,6	*-4,5*	230,7	*2,4*	246,3	*0,2*	212,7	*0,5*
**Pulses**	17,4	*7,4*	15,9	*4,2*	13,4	*5,7*	14,4	*17,0*	11,6	*-17,0*	11,8	*4,4*	13,4	*3,5*	14,5	*8,4*
**Fruits**	274,8	*9,4*	259,2	*13,7*	227,6	*13,6*	203,9	*-6,8*	222,7	*16,8*	198,1	*0,3*	254,9	*-3,0*	250,7	*10,4*
**Meat and meat products**	140,8	*4,2*	148,2	*3,0*	139,5	*5,1*	132,2	*3,6*	109,3	*-2,8*	120,9	*5,2*	131,6	*-1,4*	141,3	*-3,5*
**Fish and shellfish**	60,0	*-5,9*	68,8	*6,1*	51,1	*3,4*	52,6	*-6,9*	47,9	*-11,3*	50,8	*-0,4*	62,3	*2,1*	58,0	*-0,6*
**Alcoholic Beverages**	67,8	*-0,3*	44,9	*10,3*	102,5	*14,4*	64,0	*-10,0*	61,0	*-5,3*	68,6	*5,8*	73,1	*19,3*	56,5	*-8,5*
**Non-alcoholic Beverages**	379,9	*6,5*	300,3	*15,9*	428,2	*-5,9*	348,6	*3,1*	294,3	*-13,2*	292,8	*-1,2*	182,6	*4,0*	329,8	*-6,2*
**Sauces and condiments**	27,9	*13,5*	25,6	*12,2*	28,0	*4,5*	30,2	*13,4*	31,1	*-1,9*	31,2	*12,9*	29,7	*10,1*	30,4	*-3,7*
**Ready-to-eat-meals**	63,4	*5,2*	58,3	*34,0*	64,7	*32,9*	50,2	*16,0*	42,8	*3,3*	49,6	*8,1*	53,9	*10,9*	46,1	*4,1*
**Appetizers**	16,3	*15,1*	13,4	*3,7*	16,2	*9,5*	14,1	*-21,3*	11,2	*2,2*	11,8	*8,3*	13,6	*7,0*	12,6	*9,9*

JANUARY	EXTREMA-DURA		CASTILE AND LEON		GALICIA		ASTURIAS		CANTABRIA		BASQUE COUNTRY		LA RIOJA		NAVARRE		CANARY ISLAND	
(g/p/day)	2020	*Change (%)*	2020	*Change (%)*	2020	*Change (%)*	2020	*Change (%)*	2020	*Change (%)*	2020	*Change (%)*	2020	*Change (%)*	2020	*Change (%)*	2020	*Change (%)*
**Cereals and derivatives**	140,7	*-2,7*	183,9	*2,1*	202,4	*1,5*	184,6	*5,2*	167,9	*-5,3*	175,1	*-3,5*	159,0	*6,8*	171,1	*-0,04*	158,2	*6,3*
**Milk and dairy products**	305,6	*-11,5*	364,7	*-6,0*	338,6	*-3,5*	389,6	*2,2*	337,2	*-8,7*	336,5	*-1,0*	349,1	*28,2*	276,5	*-0,17*	315,9	*7,9*
**Eggs**	19,6	*0,0*	25,4	*10,0*	24,8	*-1,7*	27,2	*17,4*	25,5	*-8,7*	31,6	*15,9*	20,5	*-25,2*	27,0	*0,20*	22,1	*12,2*
**Sugar and sweets**	26,3	*-9,4*	24,7	*-4,9*	31,5	*3,3*	37,7	*20,6*	27,9	*2,8*	28,7	*-10,2*	32,1	*30,8*	25,9	*0,01*	29,6	*-8,3*
**Oils and fats**	22,5	*16,3*	38,0	*2,0*	41,5	*9,7*	36,8	*16,7*	39,2	*-18,0*	32,9	*16,4*	46,3	*129,1*	34,5	*0,31*	32,6	*-2,3*
**Vegetables**	223,7	*21,8*	218,4	*9,6*	212,0	*2,1*	220,4	*-7,0*	181,3	*-31,4*	272,4	*13,9*	199,3	*-0,8*	241,8	*0,19*	205,2	*-12,4*
**Pulses**	12,2	*9,1*	15,7	*23,7*	13,8	*23,1*	17,2	*3,0*	17,2	*-27,7*	18,1	*-0,6*	9,7	*-41,8*	12,7	*-0,31*	10,9	*-0,4*
**Fruits**	219,9	*7,4*	298,1	*2,0*	252,8	*-7,1*	311,0	*10,6*	236,4	*-6,5*	295,6	*-0,8*	203,5	*-2,1*	280,3	*0,09*	206,7	*-9,4*
**Meat and meat products**	113,2	*-0,9*	163,6	*8,9*	153,7	*-6,6*	130,8	*-5,5*	117,4	*-14,7*	135,9	*2,5*	144,1	*6,5*	138,9	*0,30*	99,4	*-9,1*
**Fish and shellfish**	53,6	*16,0*	73,1	*-6,6*	69,7	*-12,5*	79,8	*26,2*	49,9	*1,1*	70,0	*4,8*	58,2	*-10,0*	65,2	*0,42*	39,5	*-1,7*
**Alcoholic Beverages**	56,2	*10,0*	56,8	*20,1*	53,6	*-7,9*	53,9	*2,0*	62,8	*40,5*	55,7	*-0,8*	43,5	*-14,6*	59,9	*0,44*	61,4	*15,7*
**Non-alcoholic Beverages**	219,4	*-5,8*	233,2	*-0,7*	260,1	*-3,9*	256,8	*13,8*	275,4	*-3,2*	170,0	*-5,8*	212,6	*7,2*	177,7	*-0,13*	481,7	*0,1*
**Sauces and condiments**	28,3	*3,8*	32,6	*3,0*	25,5	*0,6*	28,7	*-5,8*	34,1	*-10,9*	31,9	*18,4*	25,6	*26,1*	24,0	*0,24*	25,6	*1,5*
**Ready-to-eat-meals**	42,0	*-3,1*	43,6	*18,3*	33,9	*5,9*	34,9	*9,3*	48,3	*12,4*	40,4	*-8,0*	52,9	*-4,5*	40,1	*0,29*	34,7	*3,1*
**Appetizers**	8,5	*-4,4*	11,0	*3,8*	9,5	*1,5*	9,3	*-1,1*	9,2	*-16,1*	13,7	*19,0*	9,9	*31,1*	14,0	*0,52*	9,5	*9,2*

FEBRUARY	CATALONIA		ARAGON		BALEARIC ISLANDS		VALENCIAN COMMUNITY		MURCIA		ANDALUSIA		COMMUNITY OF MADRID		CASTILE LA MANCHA	
(g/p/day)	2020	*Change (%)*	2020	*Change (%)*	2020	*Change (%)*	2020	*Change (%)*	2020	*Change (%)*	2020	*Change (%)*	2020	*Change (%)*	2020	*Change (%)*
**Cereals and derivatives**	177,1	9,7	148,9	6,0	218,1	14,7	175,1	4,0	163,4	-4,2	165,9	9,7	159,2	5,2	169,3	7,8
**Milk and dairy products**	292,3	2,6	313,0	16,3	299,8	-3,7	272,0	2,7	293,6	-0,8	273,0	7,7	315,7	3,5	301,2	-0,4
**Eggs**	28,2	18,1	27,3	4,9	29,1	11,6	24,2	6,7	23,6	-0,5	22,8	8,6	25,6	11,6	22,5	8,3
**Sugar and sweets**	22,5	3,4	24,9	12,4	23,8	-23,5	22,5	4,1	24,2	2,0	21,4	8,6	20,8	1,6	21,0	-8,8
**Oils and fats**	31,8	9,2	30,9	22,1	35,7	-8,2	28,6	28,8	26,9	15,1	32,3	32,4	25,1	7,0	25,6	14,4
**Vegetables**	352,5	15,0	305,7	2,1	303,3	0,9	294,3	7,0	275,7	7,7	248,6	10,9	282,5	13,2	223,5	1,6
**Pulses**	18,5	13,0	18,0	23,1	13,7	-6,6	13,2	17,4	12,9	-8,2	11,6	11,5	13,2	21,7	13,4	4,8
**Fruits**	297,0	7,2	274,0	6,2	279,6	17,8	243,9	7,7	249,9	21,2	222,1	9,0	291,4	10,1	248,1	3,0
**Meat and meat products**	145,4	4,1	153,3	3,5	140,6	1,8	137,0	3,5	120,7	-1,4	125,8	6,9	135,4	3,8	140,3	-0,4
**Fish and shellfish**	66,0	5,2	68,8	5,3	59,6	4,6	56,2	-3,1	55,7	2,9	61,7	15,2	65,8	6,1	60,7	1,6
**Alcoholic Beverages**	78,8	10,4	50,0	1,6	107,6	9,9	76,2	2,9	78,7	-2,3	78,3	8,5	75,9	8,1	71,5	14,1
**Non-alcoholic Beverages**	390,5	5,6	336,9	5,6	489,8	-6,0	376,7	9,0	384,2	-3,5	328,4	8,5	197,5	4,9	325,7	-8,4
**Sauces and condiments**	30,6	17,9	23,6	-16,6	36,4	8,1	27,2	8,6	35,4	17,4	30,6	4,4	30,4	13,4	31,1	-0,9
**Ready-to-eat-meals**	61,7	10,0	47,7	-2,8	61,0	31,5	48,2	22,4	43,0	6,9	48,3	14,7	52,2	17,9	45,8	11,1
**Appetizers**	17,3	4,3	14,7	2,5	18,9	33,0	15,1	13,8	14,8	9,3	13,5	17,5	14,8	10,5	12,9	26,4

FEBRUARY	EXTREMA-DURA		CASTILE AND LEON		GALICIA		ASTURIAS		CANTABRIA		BASQUE COUNTRY		LA RIOJA		NAVARRE		CANARY ISLAND	
(g/p/day)	2020	*Change (%)*	2020	*Change (%)*	2020	*Change (%)*	2020	*Change (%)*	2020	*Change (%)*	2020	*Change (%)*	2020	*Change (%)*	2020	*Change (%)*	2020	*Change (%)*
**Cereals and derivatives**	126,6	-11,6	184,4	0,5	203,7	3,6	197,9	11,4	185,8	-5,2	174,8	-5,5	181,3	43,0	201,7	8,4	162,4	1,5
**Milk and dairy products**	300,7	-2,0	385,5	1,8	342,5	-2,2	375,5	-1,9	348,9	-2,8	370,1	5,5	328,3	5,4	308,6	-4,5	323,0	2,8
**Eggs**	16,9	-18,6	25,8	-4,4	26,6	19,7	26,9	19,0	28,1	-5,2	32,4	22,0	27,9	15,8	30,6	11,7	22,5	-1,2
**Sugar and sweets**	17,6	-14,7	25,0	5,5	30,1	-2,4	31,1	14,8	21,3	-14,6	26,4	-7,4	29,8	34,4	32,0	29,2	27,0	2,5
**Oils and fats**	20,2	-12,4	39,6	27,3	42,5	0,4	36,8	24,5	46,3	-10,3	35,7	-0,5	21,8	-11,7	35,5	23,6	35,2	14,2
**Vegetables**	214,7	-6,9	212,9	-1,7	230,8	3,7	252,9	17,1	225,2	-13,4	315,6	23,3	256,4	18,8	260,2	7,8	237,0	3,9
**Pulses**	10,9	13,8	11,9	7,8	13,7	9,5	16,8	24,6	14,2	-10,4	17,9	20,7	13,7	-2,8	16,8	-2,6	11,3	2,1
**Fruits**	223,6	-4,9	271,4	-10,0	285,5	-4,6	339,4	16,5	274,5	-5,0	343,7	12,6	274,0	27,6	352,1	28,5	228,7	-6,7
**Meat and meat products**	113,6	4,9	153,9	-2,4	154,1	12,8	121,7	2,3	124,8	0,8	143,4	2,5	153,0	5,9	147,2	13,7	113,7	10,9
**Fish and shellfish**	50,5	-7,1	73,0	-6,4	68,3	-12,7	78,9	12,9	60,2	1,0	73,2	-2,5	71,1	4,3	63,3	14,4	37,2	-7,9
**Alcoholic Beverages**	51,6	-7,1	59,9	22,4	52,2	-0,5	55,0	-15,3	59,8	29,8	62,3	-5,4	51,3	-3,2	70,4	58,2	77,3	28,7
**Non-alcoholic Beverages**	220,8	-9,1	254,9	5,0	280,6	6,2	266,3	8,2	262,8	-0,6	175,4	-5,2	221,7	-5,4	178,0	14,0	575,4	15,1
**Sauces and condiments**	25,8	0,5	30,3	-5,8	23,0	5,2	33,9	19,9	31,9	-15,3	29,4	9,4	23,1	-4,6	21,9	-7,0	25,6	-0,2
**Ready-to-eat-meals**	36,6	-2,3	42,9	20,2	27,9	8,2	33,6	-3,1	63,9	51,5	41,4	5,8	63,0	61,2	44,3	58,8	38,5	19,2
**Appetizers**	8,7	-50,5	12,1	18,4	9,7	8,8	11,7	22,2	13,1	49,8	15,2	13,1	14,4	39,9	12,4	39,2	10,7	7,1

MARCH	CATALONIA		ARAGON		BALEARIC ISLANDS		VALENCIAN COMMUNITY		MURCIA		ANDALUSIA		COMMUNITY OF MADRID		CASTILE LA MANCHA	
(g/p/day)	2020	*Change (%)*	2020	*Change (%)*	2020	*Change (%)*	2020	*Change (%)*	2020	*Change (%)*	2020	*Change (%)*	2020	*Change (%)*	2020	*Change (%)*
**Cereals and derivatives**	191,6	17,8	167,2	20,0	254,3	39,4	204,2	18,7	193,1	24,7	195,0	28,8	187,7	30,5	199,9	32,9
**Milk and dairy products**	308,5	11,8	333,8	24,3	337,2	17,8	324,0	19,3	387,6	42,1	316,3	22,9	355,9	21,7	367,0	25,1
**Eggs**	30,9	22,6	36,8	53,7	34,6	52,2	28,9	17,5	31,4	36,0	28,0	24,2	30,4	41,1	25,4	32,2
**Sugar and sweets**	28,6	25,8	27,6	29,3	36,4	47,3	30,3	34,2	30,8	31,0	28,7	40,6	26,7	37,2	27,7	25,2
**Oils and fats**	42,7	37,8	31,6	42,3	58,2	60,7	32,4	31,8	25,8	36,0	40,2	45,7	32,2	35,9	27,5	27,4
**Vegetables**	363,5	17,3	339,6	18,5	382,7	37,9	331,8	24,1	323,0	38,3	294,3	28,0	302,2	20,7	272,0	21,9
**Pulses**	24,4	56,0	21,0	38,1	27,4	101,0	19,0	60,7	17,3	55,8	17,7	71,4	21,1	79,8	17,9	58,4
**Fruits**	312,3	10,1	311,0	13,1	326,2	37,1	261,2	5,9	251,1	27,0	252,7	18,6	303,3	12,9	268,6	14,4
**Meat and meat products**	154,4	14,0	162,3	10,6	175,2	36,7	164,2	23,4	149,7	26,1	147,5	29,7	152,6	18,2	168,5	24,6
**Fish and shellfish**	64,1	-5,2	70,9	5,9	71,1	26,3	65,3	11,1	59,2	21,8	63,6	13,5	69,2	4,5	63,4	0,8
**Alcoholic Beverages**	86,9	7,5	72,0	36,2	113,5	26,4	82,8	1,6	84,8	15,2	99,0	30,5	91,0	19,4	80,4	17,1
**Non-alcoholic Beverages**	414,3	8,1	348,9	20,7	556,0	18,5	413,1	11,4	365,9	-2,2	361,9	11,6	209,4	11,7	375,3	9,4
**Sauces and condiments**	32,3	13,3	34,4	53,2	38,3	30,8	35,7	30,4	42,0	35,9	41,2	40,1	37,5	37,7	40,4	32,6
**Ready-to-eat-meals**	66,6	21,0	52,1	29,7	65,8	50,8	53,7	30,0	52,2	39,6	53,7	38,4	57,4	32,6	52,3	40,6
**Appetizers**	18,0	8,5	15,8	22,9	18,1	28,5	18,1	25,2	18,2	44,9	15,1	27,6	17,9	25,7	14,5	22,6

MARCH	EXTREMA-DURA		CASTILE AND LEON		GALICIA		ASTURIAS		CANTABRIA		BASQUE COUNTRY		LA RIOJA		NAVARRE		CANARY ISLAND	
(g/p/day)	2020	*Change (%)*	2020	*Change (%)*	2020	*Change (%)*	2020	*Change (%)*	2020	*Change (%)*	2020	*Change (%)*	2020	*Change (%)*	2020	*Change (%)*	2020	*Change (%)*
**Cereals and derivatives**	172,6	22,5	197,7	5,7	239,3	24,3	222,4	28,5	215,2	*29,4*	204,5	16,5	177,9	26,6	210,1	22,6	220,1	43,7
**Milk and dairy products**	340,2	11,6	421,6	13,7	390,3	16,0	421,9	17,1	443,4	*36,2*	398,1	16,2	359,7	36,1	371,7	19,1	420,9	31,8
**Eggs**	26,1	27,5	30,3	18,1	30,0	7,9	28,5	30,5	34,8	*16,7*	35,0	22,2	22,3	16,4	31,6	25,8	27,0	39,9
**Sugar and sweets**	25,3	26,9	28,7	15,6	38,6	19,8	34,4	23,9	38,6	*44,5*	32,2	25,0	28,1	27,3	30,3	-12,7	37,3	43,8
**Oils and fats**	32,8	72,7	40,8	25,7	46,2	19,5	38,7	37,1	48,9	*56,1*	40,6	10,2	31,9	28,9	40,8	79,5	40,3	42,8
**Vegetables**	253,3	12,7	243,7	15,9	279,5	39,4	294,3	40,7	257,4	*16,3*	368,1	44,2	229,3	44,4	287,1	25,2	333,4	39,9
**Pulses**	16,4	79,0	18,8	48,2	14,5	18,1	19,9	48,4	33,8	*173, 5*	20,3	48,7	15,5	59,0	23,1	96,3	19,6	74,4
**Fruits**	266,5	5,8	337,2	13,7	336,3	13,3	361,0	23,1	286,5	*7,9*	349,8	15,2	263,4	24,8	314,2	20,2	300,5	15,8
**Meat and meat products**	138,9	22,4	177,6	12,9	153,8	12,3	143,0	18,9	141,1	*8,5*	153,6	15,0	159,5	40,0	167,9	35,3	158,6	42,8
**Fish and shellfish**	59,6	24,3	85,2	6,1	74,5	4,7	85,8	14,4	72,0	*31,2*	76,3	9,8	63,7	9,7	69,2	16,1	64,5	64,1
**Alcoholic Beverages**	66,0	12,8	68,5	26,3	73,6	22,3	81,2	13,9	100,2	*99,1*	85,1	32,5	70,2	35,9	80,8	42,2	87,8	46,6
**Non-alcoholic Beverages**	277,8	11,3	269,8	8,4	312,0	20,7	299,7	14,4	375,3	*28,2*	195,7	9,0	206,6	-5,6	213,2	2,8	705,6	32,9
**Sauces and condiments**	37,6	25,2	34,6	22,4	27,9	20,5	30,9	12,4	41,5	*17,8*	37,5	27,3	26,8	29,5	33,9	20,5	39,1	49,1
**Ready-to-eat-meals**	49,4	33,7	41,0	21,5	31,1	32,6	35,5	23,3	53,0	*27,4*	45,2	27,7	48,8	22,3	47,4	53,9	51,3	49,6
**Appetizers**	12,1	17,5	16,0	49,9	11,6	21,0	22,3	116,8	17,5	*79,7*	16,8	27,5	15,3	33,5	14,2	18,2	14,1	43,7

APRIL	CATALONIA		ARAGON		BALEARIC ISLANDS		VALENCIAN COMMUNITY		MURCIA		ANDALUSIA		COMMUNITY OF MADRID		CASTILE LA MANCHA	
(g/p/day)	2020	*Change (%)*	2020	*Change (%)*	2020	*Change (%)*	2020	*Change (%)*	2020	*Change (%)*	2020	*Change (%)*	2020	*Change (%)*	2020	*Change (%)*
**Cereals and derivatives**	207,9	28,4	189,7	45,5	266,9	33,8	211,7	30,6	219,1	47,5	206,3	33,9	209,8	47,3	210,5	37,2
**Milk and dairy products**	334,1	22,3	365,2	33,6	360,0	17,6	339,5	27,6	337,7	34,8	327,4	17,3	394,2	31,5	405,0	34,8
**Eggs**	38,2	52,9	43,7	71,0	40,3	61,4	39,2	59,7	32,3	37,2	35,2	51,5	39,5	79,3	31,5	53,3
**Sugar and sweets**	35,1	45,8	33,9	51,2	48,0	45,6	35,0	47,1	34,6	65,2	34,4	56,0	34,5	67,7	34,0	43,0
**Oils and fats**	38,4	31,5	47,9	69,5	49,9	3,8	30,4	27,0	30,4	18,9	43,7	40,2	34,7	38,8	31,4	17,9
**Vegetables**	410,4	49,5	422,5	60,1	487,4	77,3	372,6	49,9	356,3	44,1	343,2	43,4	378,9	59,6	304,3	38,5
**Pulses**	21,0	53,3	17,2	28,9	22,9	86,3	16,8	63,2	18,1	52,1	14,4	49,7	17,0	54,3	14,8	26,3
**Fruits**	375,9	36,0	388,5	59,0	418,9	64,3	322,0	35,3	356,6	53,3	296,6	38,4	380,6	48,6	308,0	28,2
**Meat and meat products**	173,1	33,0	187,8	33,5	175,7	27,8	175,3	40,2	146,9	38,2	157,3	36,6	182,2	44,7	177,3	39,8
**Fish and shellfish**	81,8	27,3	82,0	22,9	68,9	1,3	73,5	27,4	71,8	37,9	74,5	30,0	80,7	25,3	76,0	27,9
**Alcoholic Beverages**	131,4	64,9	134,7	128,2	162,3	23,4	115,5	44,7	132,8	62,1	137,9	81,8	136,3	80,9	116,3	65,2
**Non-alcoholic Beverages**	468,4	26,7	425,7	48,7	617,2	16,1	482,8	37,9	442,2	26,8	406,3	26,8	249,5	29,0	423,2	34,2
**Sauces and condiments**	37,6	46,5	33,2	26,5	40,2	46,1	39,4	39,4	42,1	29,6	43,4	44,9	46,3	71,9	42,2	40,5
**Ready-to-eat-meals**	68,1	25,0	54,4	47,2	55,5	18,4	53,0	33,7	49,8	25,5	49,4	21,8	59,7	39,6	49,4	20,5
**Appetizers**	25,7	51,9	23,7	88,6	26,8	66,9	22,0	14,6	22,5	78,1	19,5	57,9	25,3	86,9	20,7	69,3

APRIL	EXTREMA-DURA		CASTILE AND LEON		GALICIA		ASTURIAS		CANTABRIA		BASQUE COUNTRY		LA RIOJA		NAVARRE		CANARY ISLAND	
(g/p/day)	2020	*Change (%)*	2020	*Change (%)*	2020	*Change (%)*	2020	*Change (%)*	2020	*Change (%)*	2020	*Change (%)*	2020	*Change (%)*	2020	*Change (%)*	2020	*Change (%)*
**Cereals and derivatives**	189,2	47,2	220,7	20,9	251,1	23,5	242,1	48,1	229,2	36,5	229,2	29,5	176,5	29,8	242,1	41,4	215,4	38,8
**Milk and dairy products**	395,8	29,7	425,6	14,5	444,2	26,2	477,4	37,9	388,1	32,2	429,4	26,8	420,7	59,7	411,3	18,3	419,3	47,2
**Eggs**	26,6	40,6	38,3	38,6	38,8	53,0	43,1	107,5	40,7	64,8	46,7	72,5	35,9	78,3	49,2	109,5	33,7	58,7
**Sugar and sweets**	28,2	64,3	35,8	39,1	40,7	38,4	43,2	48,4	37,1	80,1	40,4	53,3	32,5	97,5	34,1	33,8	42,9	67,8
**Oils and fats**	30,9	41,2	44,6	20,6	48,7	27,3	43,7	61,0	45,8	49,7	40,4	14,2	28,7	9,3	34,1	18,8	47,7	60,5
**Vegetables**	311,6	58,0	297,7	35,7	323,9	45,0	336,9	75,2	276,7	10,4	400,5	52,0	267,9	19,9	363,8	59,7	385,4	68,3
**Pulses**	13,3	36,4	19,0	90,1	13,1	-4,2	15,9	46,1	19,7	31,3	19,7	60,4	12,4	57,8	14,3	36,3	14,3	37,7
**Fruits**	307,8	41,9	387,6	31,4	396,0	30,4	432,7	51,9	354,3	41,3	424,3	36,0	332,1	35,6	398,7	57,2	333,6	49,7
**Meat and meat products**	148,1	33,2	189,2	28,0	155,8	14,5	152,2	32,6	150,8	23,2	178,9	36,0	180,1	57,1	179,2	45,8	160,5	57,2
**Fish and shellfish**	73,6	35,0	85,3	17,5	101,0	36,0	99,1	44,2	77,9	13,2	103,9	38,3	72,1	1,0	81,0	30,2	65,8	30,1
**Alcoholic Beverages**	103,8	55,6	103,8	84,2	103,1	55,3	121,8	116,7	106,9	80,7	135,4	146,2	123,5	150,2	167,2	183,8	125,4	109,6
**Non-alcoholic Beverages**	317,6	26,8	319,7	31,5	330,7	21,3	327,2	26,6	311,7	16,2	253,2	61,9	229,6	14,7	220,3	20,6	650,9	21,1
**Sauces and condiments**	34,6	43,3	38,4	25,4	32,3	26,6	36,8	47,2	47,3	45,4	39,8	41,1	28,2	50,7	41,7	89,3	39,8	67,4
**Ready-to-eat-meals**	44,0	22,0	44,2	33,8	31,6	38,6	37,5	24,5	49,5	60,7	43,8	23,3	51,0	39,6	52,1	52,3	45,6	20,6
**Appetizers**	16,2	57,3	22,5	75,3	16,1	64,0	20,4	114,6	20,3	79,0	26,9	95,4	23,7	107,6	20,8	51,3	16,8	52,8

MAY	CATALONIA		ARAGON		BALEARIC ISLANDS		VALENCIAN COMMUNITY		MURCIA		ANDALUSIA		COMMUNITY OF MADRID		CASTILE LA MANCHA	
(g/p/day)	2020	*Change (%)*	2020	*Change (%)*	2020	*Change (%)*	2020	*Change (%)*	2020	*Change (%)*	2020	*Change (%)*	2020	*Change (%)*	2020	*Change (%)*
**Cereals and derivatives**	188,3	22,3	155,2	24,3	202,3	12,6	187,8	15,5	164,9	11,5	177,7	27,0	175,5	30,0	189,2	32,4
**Milk and dairy products**	312,0	6,4	334,1	24,8	343,2	13,3	313,5	14,3	304,3	22,2	304,4	17,0	358,9	24,1	356,7	13,3
**Eggs**	34,5	52,4	34,0	23,1	28,8	23,4	31,3	33,0	30,5	41,8	27,9	35,4	33,1	47,9	27,1	53,1
**Sugar and sweets**	27,1	31,6	24,8	36,1	29,7	31,1	25,4	16,6	27,4	45,0	25,0	47,2	26,4	52,2	27,5	53,1
**Oils and fats**	36,9	30,0	38,2	45,2	45,0	23,4	29,0	15,5	26,8	56,7	37,9	47,5	33,7	35,5	30,4	40,2
**Vegetables**	388,7	55,6	362,1	31,5	426,0	57,7	329,0	24,2	324,2	42,2	305,0	31,3	342,5	41,6	292,2	30,3
**Pulses**	18,5	78,8	13,8	18,9	14,8	50,1	13,1	25,8	11,0	25,3	11,7	49,4	13,4	41,8	11,5	15,1
**Fruits**	368,3	33,7	320,6	14,8	372,9	44,6	316,5	20,1	400,6	49,2	286,3	18,2	355,5	29,4	315,0	27,4
**Meat and meat products**	156,3	28,5	161,3	27,0	153,4	30,4	162,8	28,0	129,0	16,3	134,0	31,9	160,4	31,6	159,6	14,9
**Fish and shellfish**	81,9	38,2	71,4	19,0	70,7	38,9	68,9	16,0	66,6	40,3	72,6	41,3	79,4	33,6	72,5	30,4
**Alcoholic Beverages**	127,3	69,9	105,0	116,9	171,5	52,2	116,0	41,7	117,4	83,1	133,7	70,3	128,8	57,0	118,9	59,4
**Non-alcoholic Beverages**	489,8	50,0	381,1	32,4	570,4	5,8	532,7	39,7	456,9	21,5	400,2	13,1	243,9	18,4	432,6	26,3
**Sauces and condiments**	35,2	25,8	31,4	33,0	36,6	17,9	35,4	30,7	40,5	37,5	38,6	30,9	39,4	42,4	36,4	26,6
**Ready-to-eat-meals**	62,1	52,5	43,8	3,9	57,3	26,7	48,3	21,3	47,5	20,9	46,0	18,9	54,6	24,9	44,7	6,0
**Appetizers**	23,5	73,8	20,7	45,2	25,3	82,5	19,8	33,5	18,5	40,1	17,5	57,4	24,0	77,6	18,9	38,9

MAY	EXTREMA-DURA		CASTILE AND LEON		GALICIA		ASTURIAS		CANTABRIA		BASQUE COUNTRY		LA RIOJA		NAVARRE		CANARY ISLAND	
(g/p/day)	2020	*Change (%)*	2020	*Change (%)*	2020	*Change (%)*	2020	*Change (%)*	2020	*Change (%)*	2020	*Change (%)*	2020	*Change (%)*	2020	*Change (%)*	2020	*Change (%)*
**Cereals and derivatives**	179,3	43,8	202,8	18,5	212,7	11,0	198,7	13,0	190,3	-4,8	202,5	19,4	162,0	5,9	210,3	23,3	185,7	20,3
**Milk and dairy products**	411,2	35,2	429,2	21,1	371,4	12,4	425,5	6,9	350,7	11,8	372,2	10,7	338,3	24,7	350,2	-7,3	363,2	21,9
**Eggs**	24,6	48,0	34,0	44,2	30,0	28,7	28,9	44,5	36,6	24,1	38,4	32,8	32,2	35,5	37,5	93,1	30,5	30,7
**Sugar and sweets**	25,7	54,5	31,4	33,9	33,6	18,1	33,5	23,8	24,1	13,9	30,6	16,3	27,4	31,3	25,5	26,6	38,0	34,1
**Oils and fats**	33,1	75,8	43,1	10,3	45,7	3,4	40,5	22,9	47,5	74,8	41,0	28,7	57,5	69,6	34,3	19,3	40,1	13,2
**Vegetables**	291,8	42,9	291,8	31,5	279,9	14,3	270,4	24,6	264,2	18,9	368,8	41,6	284,7	42,8	370,0	64,6	305,6	23,7
**Pulses**	13,6	50,8	15,1	54,3	9,7	16,5	13,4	14,6	13,8	-8,1	14,0	6,2	12,1	27,3	12,2	-19,3	14,2	54,7
**Fruits**	324,6	45,5	388,3	23,8	335,2	3,9	358,8	9,5	292,5	19,6	398,4	20,7	288,6	18,9	374,2	34,4	293,3	13,3
**Meat and meat products**	120,8	29,7	174,6	22,8	141,9	6,4	151,2	23,7	133,5	2,0	165,0	22,2	152,3	20,8	168,8	32,5	141,8	39,0
**Fish and shellfish**	70,3	62,2	88,6	24,1	85,6	19,4	94,9	16,0	81,6	35,3	98,4	31,7	65,8	-5,2	63,6	39,9	60,6	50,0
**Alcoholic Beverages**	121,0	94,2	115,0	108,6	104,3	44,9	99,1	45,9	106,2	80,9	112,2	113,7	118,0	110,3	162,2	196,7	119,8	54,2
**Non-alcoholic Beverages**	372,8	69,4	342,2	40,0	340,6	15,6	308,7	28,8	322,2	16,0	211,0	14,1	208,7	1,1	280,8	84,9	629,5	13,8
**Sauces and condiments**	38,7	47,9	39,3	31,8	29,7	15,2	39,0	35,8	44,7	48,2	37,6	30,5	30,1	28,5	26,9	12,6	36,6	37,6
**Ready-to-eat-meals**	40,7	23,9	41,2	18,9	25,0	17,0	32,7	14,1	39,7	-6,5	37,7	5,3	34,0	-11,9	35,5	39,4	43,2	21,2
**Appetizers**	13,9	70,5	20,7	76,2	15,5	38,3	19,0	52,0	16,1	7,0	22,9	65,7	23,2	43,3	14,9	65,5	13,1	14,4

JUNE	CATALONIA		ARAGON		BALEARIC ISLANDS		VALENCIAN COMMUNITY		MURCIA		ANDALUSIA		COMMUNITY OF MADRID		CASTILE LA MANCHA	
(g/p/day)	2020	*Change (%)*	2020	*Change (%)*	2020	*Change (%)*	2020	*Change (%)*	2020	*Change (%)*	2020	*Change (%)*	2020	*Change (%)*	2020	*Change (%)*
**Cereals and derivatives**	162,4	7,6	150,0	18,1	182,7	6,0	168,3	9,3	148,2	6,0	153,7	12,3	159,3	20,9	167,3	16,3
**Milk and dairy products**	280,9	9,0	308,7	21,9	296,4	-4,0	299,9	9,8	312,8	24,9	288,9	11,1	320,0	15,2	338,1	9,8
**Eggs**	29,0	22,8	30,7	23,8	24,0	4,7	26,5	12,2	24,2	7,4	24,0	18,9	29,6	39,7	22,4	14,5
**Sugar and sweets**	23,9	27,4	20,7	30,5	28,6	47,8	21,3	15,3	21,8	14,2	20,0	34,1	20,3	32,8	21,6	18,1
**Oils and fats**	31,6	9,9	35,5	54,8	44,5	15,1	27,5	27,5	20,3	19,9	31,5	16,3	37,1	51,0	27,3	23,7
**Vegetables**	331,2	20,5	356,6	15,9	343,4	23,1	285,5	13,0	280,5	28,4	269,0	10,9	301,4	25,7	267,5	22,2
**Pulses**	15,3	16,8	12,7	7,7	13,4	17,0	12,3	27,0	10,7	35,5	9,8	14,6	12,4	32,1	9,8	6,6
**Fruits**	348,3	12,2	373,3	20,3	362,7	26,1	328,2	8,8	351,1	30,2	282,6	6,9	355,8	17,9	328,6	21,2
**Meat and meat products**	139,6	12,7	164,6	28,6	139,3	18,0	143,9	17,4	119,0	27,1	125,6	17,6	139,4	27,7	156,9	24,5
**Fish and shellfish**	72,8	18,7	77,0	16,2	56,6	0,8	64,0	7,3	57,5	7,9	63,8	13,5	73,0	23,1	68,3	19,3
**Alcoholic Beverages**	115,6	31,9	108,4	69,1	143,6	30,2	104,9	22,0	95,3	13,0	119,8	35,3	118,1	40,3	113,3	30,3
**Non-alcoholic Beverages**	451,9	2,3	374,2	-1,8	543,9	-10,8	496,6	20,2	431,8	-5,2	417,2	15,4	258,7	20,7	445,0	13,6
**Sauces and condiments**	32,1	24,6	33,0	38,0	27,6	-9,9	31,6	11,9	31,1	6,2	34,2	21,4	34,6	31,2	33,7	9,9
**Ready-to-eat-meals**	63,5	13,1	49,0	21,9	48,5	-9,9	48,1	11,6	46,7	14,8	46,5	18,8	53,0	8,1	50,8	24,1
**Appetizers**	21,5	15,7	21,5	46,8	21,2	20,1	18,5	24,8	14,5	8,9	15,3	22,7	21,2	44,5	17,1	42,8

JUNE	EXTREMA-DURA		CASTILE AND LEON		GALICIA		ASTURIAS		CANTABRIA		BASQUE COUNTRY		LA RIOJA		NAVARRE		CANARY ISLAND	
(g/p/day)	2020	*Change (%)*	2020	*Change (%)*	2020	*Change (%)*	2020	*Change (%)*	2020	*Change (%)*	2020	*Change (%)*	2020	*Change (%)*	2020	*Change (%)*	2020	*Change (%)*
**Cereals and derivatives**	149,3	14,3	181,8	13,9	197,0	5,3	197,1	20,9	179,9	8,8	182,5	6,0	153,4	15,8	174,7	9,4	173,9	16,2
**Milk and dairy products**	341,1	23,6	378,2	14,6	385,9	12,4	408,1	25,3	336,0	9,8	345,2	6,0	307,6	24,3	384,2	45,6	350,0	11,9
**Eggs**	22,7	26,3	27,0	12,1	32,4	24,2	28,7	35,7	33,0	30,0	32,6	16,8	43,5	113,6	40,8	69,2	22,9	11,7
**Sugar and sweets**	21,7	40,9	26,3	27,3	30,2	2,4	33,2	27,6	28,1	65,8	24,6	3,3	24,6	34,2	29,8	46,7	32,7	29,0
**Oils and fats**	24,2	18,2	43,5	53,5	46,4	5,9	41,7	42,3	34,8	-5,8	46,7	49,3	39,2	67,5	33,0	47,3	34,7	1,7
**Vegetables**	279,0	7,1	254,1	17,9	293,0	20,7	253,7	21,1	253,2	15,6	324,7	26,0	288,3	42,7	277,5	29,6	273,4	22,7
**Pulses**	12,6	25,0	13,4	31,1	8,8	14,3	11,4	17,8	15,1	33,0	12,3	9,2	12,7	129,9	7,7	-12,6	12,4	36,4
**Fruits**	296,8	6,7	381,1	21,3	350,0	7,8	384,6	21,4	266,0	-11,6	377,5	5,0	274,5	11,6	377,8	30,1	306,9	-4,2
**Meat and meat products**	118,4	18,6	155,3	14,3	152,7	10,7	135,6	20,9	144,4	39,4	147,5	24,5	164,9	32,8	180,2	42,7	128,7	24,2
**Fish and shellfish**	66,5	46,9	82,9	12,2	91,5	14,1	92,7	35,6	72,9	25,1	78,7	7,6	59,0	14,8	59,2	22,4	54,2	33,4
**Alcoholic Beverages**	95,8	35,2	106,2	57,5	97,0	37,4	77,1	22,4	91,5	83,1	106,6	70,1	114,5	58,3	99,9	28,4	104,1	41,5
**Non-alcoholic Beverages**	353,6	37,2	297,8	14,1	384,0	30,8	305,1	25,9	292,0	12,1	206,2	25,6	262,8	6,7	307,3	53,1	676,4	26,2
**Sauces and condiments**	32,6	20,9	37,6	48,1	28,0	13,6	36,2	32,6	38,6	31,4	33,5	29,6	28,1	19,7	30,9	21,9	34,4	19,3
**Ready-to-eat-meals**	38,9	18,5	42,1	29,3	30,4	30,4	32,2	16,8	39,1	6,2	42,3	13,3	47,1	6,4	39,8	37,4	45,5	42,4
**Appetizers**	12,7	42,7	16,8	42,1	14,3	28,2	16,8	36,1	15,3	21,8	22,0	50,8	17,7	48,2	18,6	27,7	13,9	21,0

JULY	CATALONIA		ARAGON		BALEARIC ISLANDS		VALENCIAN COMMUNITY		MURCIA		ANDALUSIA		COMMUNITY OF MADRID		CASTILE LA MANCHA	
(g/p/day)	2020	*Change (%)*	2020	*Change (%)*	2020	*Change (%)*	2020	*Change (%)*	2020	*Change (%)*	2020	*Change (%)*	2020	*Change (%)*	2020	*Change (%)*
**Cereals and derivatives**	146,6	2,2	126,0	16,8	170,0	14,2	154,2	10,5	133,8	7,1	140,9	4,3	132,9	12,8	151,1	13,8
**Milk and dairy products**	277,5	8,0	290,3	16,9	301,2	-4,4	273,3	9,7	265,0	18,8	273,6	12,9	291,5	11,6	326,4	16,1
**Eggs**	29,2	32,8	28,8	18,1	26,7	33,0	25,3	10,1	24,8	22,8	24,7	19,6	22,9	11,8	20,9	7,2
**Sugar and sweets**	18,9	28,4	17,5	11,4	22,5	39,0	17,4	26,1	18,4	27,3	15,1	2,7	15,4	9,1	18,8	22,1
**Oils and fats**	31,9	15,7	28,2	31,5	33,8	-10,7	24,4	22,5	17,7	-10,4	29,9	6,2	23,0	1,0	21,6	2,3
**Vegetables**	335,1	17,2	370,5	45,0	288,8	14,7	272,6	9,5	230,0	11,6	264,8	10,2	245,0	8,2	244,4	10,4
**Pulses**	13,1	9,8	15,1	74,3	12,4	22,7	10,6	16,4	7,8	16,5	7,7	4,6	9,3	8,9	9,9	31,6
**Fruits**	374,7	11,4	354,3	18,6	358,4	1,2	347,5	8,4	336,2	17,0	302,9	10,3	318,3	4,3	324,1	20,3
**Meat and meat products**	124,2	12,2	144,6	31,5	129,6	23,6	129,3	20,1	108,0	17,5	110,1	8,2	114,1	8,2	141,1	26,9
**Fish and shellfish**	68,9	11,5	68,1	14,0	60,4	8,0	63,7	14,5	52,9	20,8	63,0	9,9	60,6	9,6	68,6	34,4
**Alcoholic Beverages**	109,9	22,3	90,6	51,6	159,4	24,8	112,6	30,4	115,4	51,4	127,7	44,6	108,3	40,2	119,3	41,9
**Non-alcoholic Beverages**	496,9	4,2	342,4	-4,0	711,5	-3,9	515,8	15,1	402,0	-12,3	438,9	17,3	271,5	13,3	487,7	-0,3
**Sauces and condiments**	29,0	10,3	28,2	14,8	33,4	12,1	30,8	18,4	30,8	-2,1	31,3	16,9	30,8	19,0	30,2	2,6
**Ready-to-eat-meals**	61,6	11,3	45,8	5,4	60,1	30,3	47,5	8,0	46,6	16,8	43,2	13,0	52,1	7,5	45,8	13,0
**Appetizers**	21,6	13,0	17,5	-0,3	21,9	26,0	17,6	18,8	14,4	13,1	15,2	20,6	17,2	21,5	16,4	20,4

JULY	EXTREMA-DURA		CASTILE AND LEON		GALICIA		ASTURIAS		CANTABRIA		BASQUE COUNTRY		LA RIOJA		NAVARRE		CANARY ISLAND	
(g/p/day)	2020	*Change (%)*	2020	*Change (%)*	2020	*Change (%)*	2020	*Change (%)*	2020	*Change (%)*	2020	*Change (%)*	2020	*Change (%)*	2020	*Change (%)*	2020	*Change (%)*
**Cereals and derivatives**	136,6	1,8	159,6	-2,6	184,1	1,0	203,1	26,8	177,8	2,7	149,5	-4,3	133,4	7,3	161,3	-17,6	149,8	5,5
**Milk and dairy products**	346,1	12,5	341,3	2,6	371,5	13,9	392,5	13,5	316,3	0,5	323,7	6,5	276,2	-6,6	315,3	-6,9	320,8	6,7
**Eggs**	23,4	22,4	23,6	-10,8	31,0	22,3	26,7	21,8	28,0	-8,2	30,8	25,1	31,5	66,7	25,4	9,7	20,8	-12,6
**Sugar and sweets**	15,5	-4,0	19,9	6,6	27,2	17,3	22,3	-41,4	24,1	28,1	22,9	19,1	22,7	33,7	21,1	8,6	28,2	27,8
**Oils and fats**	24,5	3,0	31,2	-6,4	41,7	21,3	33,3	21,6	33,5	10,1	38,8	33,6	30,9	4,5	32,9	4,6	28,4	39,6
**Vegetables**	277,7	-18,5	273,1	-4,9	332,6	-4,3	262,1	25,9	277,1	18,3	296,8	29,2	243,1	17,7	342,8	-30,1	219,9	3,3
**Pulses**	9,0	-41,7	9,4	14,0	7,6	25,9	12,0	31,8	13,1	15,4	11,2	38,3	7,6	14,6	9,7	13,9	8,6	0,9
**Fruits**	331,2	5,2	370,5	5,5	375,6	14,3	392,4	19,3	309,2	8,4	346,5	-1,3	304,9	10,8	361,1	-6,1	292,2	-7,7
**Meat and meat products**	103,5	-3,9	147,1	8,9	133,1	8,7	129,4	22,6	134,1	19,6	129,0	15,3	121,3	19,8	140,7	13,6	118,3	18,9
**Fish and shellfish**	57,2	9,3	76,6	12,0	93,1	11,8	88,9	26,0	70,5	6,8	75,0	19,7	54,7	14,3	59,9	9,0	42,2	-8,2
**Alcoholic Beverages**	118,1	44,2	92,6	22,0	96,2	28,5	84,2	62,8	87,0	22,0	93,6	58,8	84,1	13,9	94,7	80,7	90,3	18,1
**Non-alcoholic Beverages**	422,7	22,5	305,8	3,8	350,0	24,0	330,7	21,3	276,1	-12,8	160,8	-7,6	261,1	25,0	216,8	-10,2	582,5	4,5
**Sauces and condiments**	32,3	12,9	30,7	5,0	27,0	2,2	29,9	-4,2	35,8	3,2	29,4	4,6	19,8	12,4	24,4	-13,3	29,4	11,6
**Ready-to-eat-meals**	34,1	8,6	38,2	1,4	27,5	14,6	30,6	24,5	42,6	37,7	32,6	-4,7	39,8	-6,4	31,3	3,0	40,6	2,5
**Appetizers**	12,5	40,5	17,4	21,5	14,1	19,7	14,8	11,7	17,7	-9,6	17,3	11,3	13,6	21,1	14,9	27,5	11,3	14,4

AUGUST	CATALONIA		ARAGON		BALEARIC ISLANDS		VALENCIAN COMMUNITY		MURCIA		ANDALUSIA		COMMUNITY OF MADRID		CASTILE LA MANCHA	
(g/p/day)	2020	*Change (%)*	2020	*Change (%)*	2020	*Change (%)*	2020	*Change (%)*	2020	*Change (%)*	2020	*Change (%)*	2020	*Change (%)*	2020	*Change (%)*
**Cereals and derivatives**	132,8	-0,4	121,7	2,3	162,2	5,8	143,7	1,5	127,5	-1,3	134,3	0,0	116,8	9,1	134,5	3,1
**Milk and dairy products**	242,6	3,0	261,1	11,4	287,8	-6,1	248,9	-1,4	254,6	-3,2	257,3	5,0	256,2	3,2	284,9	6,3
**Eggs**	24,3	13,5	30,6	28,8	25,0	12,6	25,6	14,8	21,7	18,4	22,1	4,8	21,5	12,4	20,8	15,2
**Sugar and sweets**	16,7	2,5	17,2	11,4	18,8	-14,4	16,9	9,0	17,8	25,9	15,4	6,9	14,5	8,5	12,9	-42,0
**Oils and fats**	30,8	33,3	23,6	19,2	40,0	10,5	21,0	17,1	21,1	-17,0	30,4	10,7	20,0	-5,6	22,5	4,8
**Vegetables**	281,5	-1,6	293,2	5,2	307,4	3,1	253,5	3,2	218,5	10,4	232,6	5,7	210,0	6,3	211,0	10,4
**Pulses**	12,1	13,5	11,7	39,2	15,0	5,6	10,6	36,9	9,0	21,7	8,4	9,8	8,6	10,8	9,1	5,4
**Fruits**	332,2	9,8	343,1	25,6	372,3	3,5	308,2	10,0	300,1	23,3	269,2	7,2	256,0	1,0	260,5	11,8
**Meat and meat products**	111,4	4,0	124,6	15,1	129,3	14,7	114,9	5,7	94,4	10,1	106,2	4,0	99,5	4,5	129,7	2,7
**Fish and shellfish**	62,2	5,4	58,3	-3,2	53,1	-0,9	58,5	14,8	53,2	14,7	56,2	8,6	51,3	2,5	55,2	2,1
**Alcoholic Beverages**	98,6	19,8	85,1	55,0	132,6	9,9	102,6	17,9	95,0	-17,0	110,0	12,6	82,9	11,1	86,2	13,8
**Non-alcoholic Beverages**	462,9	2,9	327,7	-5,8	726,1	4,5	493,6	14,4	456,3	5,9	424,0	11,6	253,0	19,1	398,3	12,7
**Sauces and condiments**	26,0	1,6	24,4	15,5	32,3	14,3	29,4	18,5	30,8	11,1	30,6	3,9	25,0	2,9	27,9	3,8
**Ready-to-eat-meals**	52,1	-1,5	36,4	-3,8	49,0	2,9	42,6	5,4	47,1	9,2	40,6	7,3	42,0	-3,9	37,5	-1,8
**Appetizers**	19,2	13,7	14,8	1,2	20,9	7,2	15,7	4,9	14,6	0,7	12,7	2,0	14,9	16,0	13,9	6,8

AUGUST	EXTREMA-DURA		CASTILE AND LEON		GALICIA		ASTURIAS		CANTABRIA		BASQUE COUNTRY		LA RIOJA		NAVARRE		CANARY ISLAND	
(g/p/day)	2020	*Change (%)*	2020	*Change (%)*	2020	*Change (%)*	2020	*Change (%)*	2020	*Change (%)*	2020	*Change (%)*	2020	*Change (%)*	2020	*Change (%)*	2020	*Change (%)*
**Cereals and derivatives**	117,8	-7,7	153,2	-4,6	183,6	5,8	176,0	12,3	143,7	3,9	164,9	20,8	136,7	20,4	167,0	9,3	139,3	0,9
**Milk and dairy products**	289,0	-9,7	343,4	2,1	318,9	8,9	333,0	0,1	352,3	2,8	300,4	2,8	292,6	4,6	320,3	11,8	260,7	-11,3
**Eggs**	19,9	-8,4	25,0	-0,9	28,3	27,6	28,7	24,7	28,3	14,9	26,6	4,9	32,7	47,2	24,3	-8,2	19,5	-3,4
**Sugar and sweets**	17,1	4,7	17,8	-15,9	27,7	7,8	29,8	11,7	20,7	-4,8	18,3	-1,6	22,6	37,5	13,4	-27,5	22,8	4,1
**Oils and fats**	22,5	-17,9	40,8	42,3	40,9	-7,6	34,5	7,1	45,8	45,8	35,1	38,0	26,1	-27,3	39,6	20,9	25,1	2,6
**Vegetables**	233,7	-23,4	256,0	-5,3	278,3	-11,8	233,5	7,6	223,8	-13,2	269,8	13,8	274,7	41,2	398,5	71,1	194,6	-5,3
**Pulses**	8,6	-4,3	9,5	35,5	9,0	22,3	11,3	14,0	8,8	-19,2	9,4	38,0	6,7	-35,2	6,4	-1,7	9,3	16,4
**Fruits**	257,6	-13,9	350,8	5,6	340,9	7,7	345,5	13,4	311,8	29,5	317,1	5,4	363,7	46,8	359,7	37,2	251,1	-9,6
**Meat and meat products**	103,2	-3,5	129,9	-6,1	126,2	7,1	119,0	15,7	126,0	-6,3	121,3	20,1	104,5	9,4	126,4	22,8	99,0	8,4
**Fish and shellfish**	47,9	-6,6	70,9	6,2	82,5	13,0	78,7	16,2	58,0	3,9	70,7	25,9	56,1	15,0	63,4	22,0	43,4	9,5
**Alcoholic Beverages**	94,3	23,0	85,0	33,9	82,7	21,2	78,0	30,8	89,8	12,1	91,5	53,4	99,3	103,2	107,0	62,5	85,1	43,0
**Non-alcoholic Beverages**	355,0	14,6	320,8	24,0	329,3	18,6	309,5	30,0	295,5	-14,7	167,5	3,1	226,2	-5,1	224,7	-7,7	599,2	2,6
**Sauces and condiments**	27,3	2,9	30,1	4,4	27,3	34,3	29,6	-3,3	35,2	14,2	29,0	20,4	22,6	15,2	31,9	8,5	28,6	8,0
**Ready-to-eat-meals**	36,3	-0,4	31,9	-13,2	24,2	28,4	26,3	2,4	36,4	-0,1	32,3	-4,8	47,7	39,9	40,8	37,8	37,4	9,2
**Appetizers**	10,7	-2,3	13,9	7,4	12,6	16,5	12,9	16,6	11,8	-18,2	16,1	37,2	14,6	67,1	12,0	13,9	14,0	34,0

Table S2. Energy intake by regions. Year 2020 and change versus 2019.

	JAN		FEB		MAR		APR		MAY		JUN		JUL		AUG	
**REGIONS (kcal/p/day)**	2020	*Change (%)*	2020	*Change (%)*	2020	*Change (%)*	2020	*Change (%)*	2020	*Change (%)*	2020	*Change (%)*	2020	*Change (%)*	2020	*Change (%)*
**ANDALUSIA**	1.936	*6,3*	2.080	*12,2*	2.481	*31,3*	2.700	*36,7*	2.361	*32,0*	2.105	*15,3*	1.955	*9,1*	1.856	*4,9*
**ARAGON**	2.073	*11,9*	2.106	*6,4*	2.345	*25,3*	2.838	*49,4*	2.335	*29,0*	2.278	*26,4*	2.008	*20,4*	1.783	*8,9*
**ASTURIAS**	2.310	*7,4*	2.331	*11,5*	2.632	*28,3*	2.994	*52,2*	2.574	*19,1*	2.486	*25,1*	2.351	*18,7*	2.182	*9,4*
**BALEARIC ISLANDS**	2.404	*12,5*	2.524	*5,8*	3.185	*42,4*	3.355	*30,3*	2.853	*27,8*	2.516	*10,5*	2.385	*10,8*	2.347	*4,2*
**CANARY ISLAND**	2.004	*1,6*	2.169	*7,1*	2.850	*42,1*	2.979	*49,3*	2.587	*24,1*	2.428	*19,5*	2.092	*11,4*	1.874	*2,1*
**CANTABRIA**	2.157	*-9,3*	2.373	*-2,4*	2.848	*35,0*	2.824	*37,5*	2.477	*13,2*	2.335	*14,9*	2.298	*10,1*	2.156	*7,3*
**CATALONIA**	2.175	*4,3*	2.317	*8,3*	2.597	*19,5*	2.851	*34,8*	2.610	*22,5*	2.318	*13,1*	2.181	*10,6*	1.967	*7,4*
**CASTILE LA MANCHA**	1.977	*-5,6*	2.040	*5,3*	2.403	*26,9*	2.637	*35,0*	2.395	*26,8*	2.235	*20,0*	2.031	*15,8*	1.775	*3,0*
**VALENCIAN COMMUNITY**	2.051	*6,1*	2.134	*8,3*	2.509	*24,0*	2.679	*36,1*	2.419	*21,8*	2.214	*14,4*	2.062	*13,8*	1.888	*6,7*
**CASTILE AND LEON**	2.278	*3,7*	2.276	*4,7*	2.535	*15,6*	2.840	*27,9*	2.672	*23,6*	2.451	*24,0*	2.142	*2,2*	2.087	*5,0*
**EXTREMADURA**	1.814	*-0,3*	1.681	*-8,3*	2.236	*25,7*	2.441	*42,6*	2.308	*44,9*	2.007	*19,7*	1.890	*2,3*	1.706	*-5,1*
**GALICIA**	2.354	*1,0*	2.370	*3,2*	2.705	*21,3*	2.952	*28,0*	2.574	*13,5*	2.536	*9,5*	2.406	*9,9*	2.243	*4,7*
**COMMUNITY OF MADRID**	1.989	*4,1*	2.076	*8,3*	2.434	*28,0*	2.807	*47,0*	2.507	*35,1*	2.294	*26,1*	1.878	*10,2*	1.629	*4,6*
**MURCIA**	1.827	*-0,1*	2.050	*2,3*	2.403	*29,7*	2.710	*43,2*	2.262	*27,8*	1.993	*14,4*	1.816	*10,6*	1.764	*1,0*
**NAVARRE**	2.099	*11,1*	2.318	*13,5*	2.561	*30,8*	2.845	*41,2*	2.495	*28,8*	2.368	*30,4*	2.099	*-5,1*	2.170	*18,8*
**BASQUE COUNTRY**	2.199	*3,1*	2.307	*1,4*	2.609	*18,6*	2.976	*36,8*	2.672	*25,1*	2.493	*19,4*	2.154	*12,8*	2.047	*17,6*
**LA RIOJA**	2.154	*17,6*	2.138	*18,5*	2.219	*30,6*	2.436	*40,7*	2.404	*25,4*	2.267	*32,6*	1.850	*7,9*	1.886	*15,7*
